# Cancer-associated fibroblast-derived extracellular vesicles facilitate metastasis in hepatocellular carcinoma by delivering CTGF

**DOI:** 10.1007/s13402-025-01085-2

**Published:** 2025-07-01

**Authors:** Mengli Zheng, Luyao Liu, Haochen Cui, Yuchong Zhao, Wei Chen, Shuya Bai, Wang Peng, Yun Wang, Yanling Li, Ronghua Wang, Xiju Wang, Bin Cheng

**Affiliations:** 1 Department of Gastroenterology and Hepatology, Tongji Hospital, Tongji Medical College, Huazhong University of Science and Technology, Wuhan, 430030 China; 2 Department of Digestive Endoscopy, The Affiliated Hospital of Guizhou Medical University, Guiyi Street No. 28, Guiyang, Guizhou, China 550000; 3 Department of Gastroenterology, The Tenth Affiliated Hospital of Southern Medical University (Dongguan People’s Hospital), Southern Medical University, Dongguan, 523000 Guangdong Province China; 4 Department of Surgery, University of Pittsburgh School of Medicine, Pittsburgh, PA 15213 USA

**Keywords:** Hepatocellular carcinoma, Metastasis, Extracellular vesicles, Cancer-associated fibroblasts, Tumor microenvironment, CTGF

## Abstract

**Purpose:**

The tumor microenvironment (TME) plays a crucial role in cancer progression. Cancer-associated fibroblasts (CAFs) are key components of the TME and play critical roles in tumor development and metastasis. However, the mechanisms by which CAFs influence hepatocellular carcinoma (HCC) metastasis are not fully understood.

**Methods:**

Extracellular vesicles (EVs) from CAFs and normal fibroblasts (NFs) were characterized via western blotting, transmission electron microscopy, and nanoparticle tracking analysis. An iTRAQ-based proteomic sequencing analysis was conducted to quantify proteins in the EVs from these cells. Colony formation assays and Transwell assays were used to assess tumor cell proliferation and migration. Xenograft tumor models were established in nude mice to evaluate tumor progression *in vivo*. Coimmunoprecipitation and molecular docking were performed to explore the interactions between CTGF and Notch1.

**Results:**

A high CAF abundance is associated with poor prognosis in HCC patients. EVs from CAFs significantly enhanced the proliferative and invasive abilities of HCC cells *in vitro* and *in vivo*. Connective tissue growth factor (CTGF) was found to be highly upregulated in CAF-derived EVs, and CTGF knockdown in CAF-derived EVs attenuated their tumor-promoting capacities. Mechanistically, CTGF derived from CAF-EVs activated the Notch1/Snail1 signaling pathway in recipient cells via interaction with the Notch1 receptor, enhancing HCC cell proliferation and invasion. Furthermore, high CTGF expression was significantly correlated with poor clinicopathological features in HCC patients.

**Conclusion:**

Our findings revealed that CTGF derived from CAF-EVs promoted the proliferation and invasion of HCC cells via activation of the Notch1/Snail1 pathway, highlighting CTGF derived from CAF-EVs as a prognostic biomarker and therapeutic target in HCC.

**Supplementary information:**

The online version contains supplementary material available at 10.1007/s13402-025-01085-2.

## Introduction

Hepatocellular carcinoma (HCC) is the sixth most common malignancy and the fourth leading cause of cancer-related death worldwide [1]. Despite significant progress in diagnosis and treatment over the past decades, preventing cancer recurrence and metastasis remains a major challenge [2]. Recently, an increasing number of studies have demonstrated that the tumor microenvironment (TME), which consists of various cell types, soluble factors, signaling molecules, and extracellular matrix (ECM) components, contributes to tumor development, metastasis, relapse, and drug resistance [3]. Normal fibroblasts (NFs) can be educated and transformed into activated cancer-associated fibroblasts (CAFs), which facilitate tumor development and progression [4, 5]. CAFs, the primary component of the TME stroma, are crucial for tumor proliferation, invasion, and relapse through the secretion of soluble factors and extracellular vesicles (EVs) [6–8]. CAFs release various soluble factors, such as periostin, PDGF, and SPP1, promoting tumor lymphatic metastasis and drug resistance [9–11]. Our previous studies also revealed that CAFs promote HCC cell stemness via the Notch1 signaling pathway through the paracrine effects of IL-6 and STC1 [6, 12]. Additionally, targeting CAFs and their secreted factors could activate immune cell-mediated antitumor immunity and reduce tumor metastasis [13, 14].

Within the TME, CAFs constitute a heterogeneous population exhibiting substantial phenotypic and functional diversity. Research has identified several distinct CAF subpopulations with specialized characteristics and functions [15, 16]. Myofibroblastic CAFs (myCAFs) express high levels of alpha-smooth muscle actin (α-SMA) and primarily contribute to ECM remodeling. Inflammatory CAFs (iCAFs) secrete pro-inflammatory cytokines, including IL-6 and IL-8, while antigen-presenting CAFs (apCAFs) express MHC-II molecules and participate in immune regulation. These subpopulations demonstrate unique gene expression signatures, distinct spatial organization within tumors, and specialized functional roles in the TME. Notably, myCAFs tend to reside in close proximity to cancer cells, whereas iCAFs typically reside at greater distances from tumor cells and predominantly orchestrate immunomodulatory responses [17]. This functional heterogeneity significantly influences tumor progression, immune surveillance, and therapeutic responsiveness.

Emerging evidence revealed that EVs serve as crucial mediators for intercellular communication and are extensively involved in the interaction profile between cancer cells and tumor stroma [18–20]. Crosstalk between CAFs and tumor cells via EVs can facilitate TME remodeling to promote tumor metastasis and treatment resistance [21, 22]. For example, EVs from HCC cells contribute to CAF activation, which further promotes lung metastasis [23]. Conversely, CAF-derived EVs promote tumor proliferation, invasion, and cell stemness [21, 24]. In lung adenocarcinoma, the CAF-specific lncRNA LINC01614 enhances glutamine uptake and cancer cell progression [25]. Similarly, CAF-derived EVs with high expression of miR-3173-5p induce chemoresistance in pancreatic cancer cells [26]. Despite these findings, the effects of proteins derived from CAF-EVs on HCC progression remain largely unknown.

In this study, our mass spectrometry analysis and subsequent validation revealed that connective tissue growth factor (CTGF) was highly expressed in CAF-derived EVs. We demonstrated that CTGF derived from CAF-EVs was directly transferred to HCC cells, triggering the Notch1/Snail1 signaling pathway and thereby promoting tumor growth and metastasis. Our data shed new light on the impact of CAFs on tumor progression via EVs, highlighting CTGF as a potential therapeutic target for HCC.

## Methods

### Patients and samples

47 HCC tissue samples, 4 pairs of HCC tissues, and the corresponding adjacent normal liver tissues were collected from HCC patients treated at Tongji Hospital, Huazhong University of Science and Technology (HUST, Wuhan, China). Patients who had received radiotherapy and chemotherapy were removed. This study was approved by the ethics committee of Tongji Hospital, HUST, Wuhan, China (IRB ID: TJ-IRB20220970).

### Isolation of CAFs and NFs

CAFs and NFs were isolated from four pairs of collected HCC specimens and adjacent normal liver tissues, respectively. The isolation of CAFs and NFs was performed as described previously [12]. Briefly, fresh HCC tissues and adjacent normal tissues were washed with D-Hank’s solution (Servicebio), cut into 2–3 mm fragments, and plated in 6-well plates with DMEM (GIBCO, Cat#11,965,092) containing 15% FBS (GIBCO, Cat#MT35010CV) to allow attachment. CAFs and NFs were allowed to grow out of the tumor pieces for 7–14 days. After 2–3 generations, 95% of the CAFs and NFs were purified and identified by the expression of α-SMA (also known as ACTA2), fibroblast activation protein (FAP), and vimentin.

### Cell lines and culture

The HCC cell lines MHCC-97 H and SNU-398 were purchased from the Cell Bank of the Chinese Academy of Sciences (Shanghai, China) and the American Type Culture Collection (ATCC, Manassas, VA, USA), respectively. CAFs, NFs, and the HCC cell lines MHCC-97 H and SNU-398 were cultivated in DMEM. All mediums comprised 10% FBS and 1% penicillin-streptomycin. All the cells were maintained in a 5% CO_2_ incubator at 37℃.

### Preparation of conditioned medium (CM)

CAFs and NFs were plated in 6-well plates (1 × 10^5^ cells/well) and allowed to attach for 24 hours. CAFs and NFs were washed twice with PBS (Servicebio, .Cat#G4202), and the medium was replaced with 2 mL of serum-free DMEM per well. For the transfected cells, the culture medium was replaced with serum-free DMEM 48 hours post-transfection. Subsequently, the CAFs and NFs were incubated for 24 hours at 37 °C. The CM was subsequently collected, filtered through membrane syringe filters (0.22 μm) to remove cellular debris, and stored at − 80 °C for subsequent experiments.

### Cell treatment

MHCC-97 H and SNU-398 cells were maintained in DMEM supplemented with 10% FBS for 24 hours before CM or EV treatment. For CM treatment, HCC cells were incubated with a 1:1 ratio (v/v) of fresh culture medium and CM (either CAF-CM or NF-CM) for 48 hours for experiments. Control cells received a 1:1 ratio (v/v) of fresh complete culture medium and serum-free DMEM. For EV treatment, HCC cells were treated with EVs (50 μg/mL of total protein) isolated from either CAFs or NFs for 48 hours. Then, colony formation assay, Transwell assays, and western blot assays were carried out.

### Immunofluorescence (IF)

The cells were fixed with 4% paraformaldehyde for 20 min, washed with PBS, and permeabilized with 0.1% Triton X-100 (Servicebio, Cat#G3068-100ML) for 10 min. After being washed with PBS for 3 times, the cells were blocked with 10% goat serum for 40 min. Then, the cells were incubated with primary antibodies in PBS at 4 °C overnight. The stained cells were imaged via a fluorescence microscope (OLYMPUS). The antibodies used are presented in Table S1.

### Immunohistochemistry (IHC)

To assess protein expression levels, IHC assays using antibodies against CTGF and Snail1 were performed according to the protocol described previously [27, 28]. Briefly, Tumor tissues were fixed in 4% paraformaldehyde, dehydrated, and paraffin-embedded. Sections (4 μm) were deparaffinized in xylene and rehydrated through graded alcohols. Antigen retrieval was performed in Tris-EDTA buffer (Proteintech, Cat#PR30002) at 95 °C for 20 min. After cooling, sections were treated with 3% hydrogen peroxide (BOSTER, Cat#AR1108) for 15 min and blocked with 10% goat serum. Primary antibodies were applied overnight at 4 °C. Following PBS washes, sections were incubated with HRP-conjugated secondary antibodies for 1 h. Immunoreactivity was visualized using a diaminobenzidine (DAB) kit (Proteintech, Cat#PR30010). The antibody details are provided in **Table S1**. We quantified the staining intensity via the ImageJ IHC profiler on a four-point scale: 0 (negative, no staining), 1 (weakly positive, light yellow), 2 (moderately positive, brown), and 3 (strongly positive, brown-red). We evaluated the extent of staining on the basis of the percentage of positively stained cells relative to the total area and assigned scores ranging from 0 to 4: 0 (0%), 1 (1–25%), 2 (26–50%), 3 (51–75%), and 4 (76–100%). Protein expression (ranging from 0 to 12) was assessed by multiplying the staining intensity by the extent of staining. For survival analysis, we stratified patients into low (≤1 grade) and high (>1 grade) expression groups. Two pathologists, blinded to the clinical data, independently reviewed the IHC results. Representative images illustrating various staining intensities are presented in Fig. S4a-c.

### EV extraction, characterization, and quantification

CAFs and NFs were maintained in DMEM supplemented with 10% FBS depleted of EVs by ultracentrifugation at 100,000 g at 4℃ for 12 h [29]. After 48 h, EVs from CAF-CM and NF-CM were collected via differential centrifugation. To remove dead cells, CAF-CM and NF-CM were centrifuged at 800 × g for 10 min and 3000 × g for 30 min at 4 °C. The supernatant was subsequently centrifuged at 10,000 × g for 70 min and 100,000 × g for 70 min. Then, the deposited debris was washed twice with PBS and centrifuged at 100,000 × g for 70 min at 4 °C. Finally, the deposited debris was resuspended in PBS and stored at − 80 °C.

The characterization of the EVs was performed as described previously [30]. Briefly, the expression of EV markers was validated by western blot, and the size and morphology of the EVs were analyzed by transmission electron microscope (TEM) and nanoparticle tracking analysis (NTA). The antibodies used are listed in **Table S1.** For EV quantification, the debris was resuspended in PBS, and total protein content was measured by mixing 200 µL Pierce BCA Protein Assay Kit (Thermo Fisher Scientific, Cat#23,225) with 1–5 µL EV samples. Resulting alterations in solution absorbance were quantified at 562 nm according to the manufacturer’s instructions following incubation for 30 min at 37 °C.

### EV labeling and tracing

The EV tracing assay was carried out as described previously [30]. Briefly, the EVs were incubated with PKH67 Green Fluorescent Cell Linker Kit (Sigma Aldrich, St Louis, USA, Cat#MINI67) for 5 min, and then the staining was stopped by the addition of serum. PKH67-labeled EV pellets were washed twice with PBS, collected by ultracentrifugation, and resuspended in PBS. The amount of EV protein was assessed by the BCA assay and adjusted to a concentration of 1 mg/mL with PBS. Then, PKH67-labelled EVs (50 μg/mL of total protein) were resuspended in culture medium containing 10% EV-free FBS, filtered through membrane syringe filters (0.22 μm), and subsequently added to MHCC-97 H and SNU398 cells seeded in 24-well plates for 24 h in 5% CO_2_ at 37 °C. After washing twice with PBS, cells were fixed with 4% formaldehyde and stained with DAPI (Servicebio, Cat#G1012). Phalloidin (Beyotime, Cat#12,352,207) was used to label F-actin in HCC cells. The images were obtained using a Zeiss confocal microscope system (LSM710, Zeiss, Pleasanton, CA, USA).

### Viral infection

Lentiviruses expressing CTGF shRNA, CTGF, Notch1 shRNA, and Notch1 were purchased from Genechem Corporation (Shanghai, China). The sequences used for lentivirus-based RNAi are listed in Table S2. CAFs, NFs, and HCC cells were infected with lentivirus in the presence of polybrene (Sigma‒Aldrich, Cat#TR-1003-G) for 48 h. Puromycin (MCE, Cat#HY-B1743A) was used to select transduced cells for 7 days.

### Colony formation assay

The cells were plated at a density of 2 × 10^3^ cells per well in 6-well plates. After 14 days, the colonies were fixed with 4% methanol, dyed with crystal violet solution, and counted via a light microscope (OLYMPUS). Three independent experiments were carried out.

### Transwell migration and invasion assays

Cell migration and invasion were analyzed using Transwell chambers (8-μm pore size; Millipore, Billerica, MA, USA) without or with Matrigel matrix (Corning, Cat#356,234). A total of 5 × 10^4^ cells were cultured with FBS-free culture medium in the upper chamber, while the bottom chamber was filled with 10% FBS culture medium. After 28 h (migration) or 32 h (invasion), the bottom surface of the chambers was washed with PBS, fixed with 4% methanol, dyed with crystal violet solution, and imaged via a light microscope (OLYMPUS).

### Western blot

Western blot was carried out as described previously [30]. Briefly, total cellular protein was extracted using RIPA lysis buffer (Wuhan Promotor Biological, Cat#B1025) containing protease inhibitor cocktail (MCE, Cat#HY-K0021) and phenylmethylsulfonyl fluoride (MCE, Cat#HY-B0496). Protein concentration was determined using a BCA assay kit. Protein samples were boiled with 5 × SDS-PAGE loading buffer (Servicebio, Cat#G2013) at 95 °C for 10 min. Equal amounts of protein were separated by 10% SDS-polyacrylamide gel electrophoresis and transferred onto PVDF membranes (0.45 μm, Millipore, Cat#IPVH00010). Membranes were blocked with 5% nonfat milk in TBST for 1 h at room temperature, then incubated with primary antibodies overnight at 4 °C. After washing with TBST, membranes were incubated with HRP-conjugated secondary antibodies for 1 h at room temperature. Protein bands were visualized using an enhanced chemiluminescence detection kit (Abbkine, Cat#BMU102-EN). The primary antibodies used are listed in Table S1. Polyclonal rabbit anti-β-actin was used as an internal control.

### Mouse and tumor models

Six-week-old male BALB/c nude mice were purchased from Beijing Vital River Laboratory Animal Technology Co., Ltd. and raised under pathogen-free conditions. Animal experiments were carried out according to the NIH Guide for the Care and Use of Laboratory Animals and were approved by the Tongji Hospital Institutional Review Board (IACUC number: 3735). For the orthotopic liver xenograft tumor models, luciferase-labeled MHCC-97 H cells (1 × 10^6^) suspended in 100 μL of PBS were injected into the left lobe of the liver. A total of 10 μg of CAF-EVs, NF-EVs, or PBS was injected every three days through the tail vein. Bioluminescence was assessed 5 weeks postinjection, 5 minutes after the administration of 100 μL of potassium D-luciferin salt (30 mg/mL in PBS, MCE, Cat#HY-12591B) via intraperitoneal injection. After 6 weeks, the mice were anesthetized, and the lungs were collected, fixed with formalin, and paraffin-embedded for hematoxylin and eosin (H&E) staining.

### LC-MS/MS analysis

NF-EVs and CAF-EVs were used for proteomics analysis. Proteins derived from NF-EVs and CAF-EVs were extracted, digested with trypsin (YEASEN, Cat#40127ES60), and labeled using the iTRAQ Reagent kit (Sigma-Aldrich, Cat#4,381,663). LC-MS/MS analysis was performed on an Orbitrap mass spectrometer. Data were processed using Proteome Discoverer against the human UniProt database with a false discovery rate of < 1%. Differentially expressed proteins were identified using thresholds of P-value < 0.05 and fold change ≥ 1.2. The proteomics analysis was performed by Omicsolution Co., Ltd (Shanghai, China). The data of differentially expressed proteins between NF-EVs and CAF-EVs identified by proteomics analysis were provided in Table S3.

### Coimmunoprecipitation (Co-IP)

Co-IP was performed as described previously [31]. Briefly, after incubation with CAF-EVs for 24 h, MHCC-97 H and SNU-398 cells were lysed with RIPA lysis buffer (Wuhan Promotor Biological, Cat#B1025) containing a protease inhibitor cocktail (MCE, Cat#HY-K0021). The cell extracts were incubated with CTGF or Notch1 antibodies overnight at 4 °C, followed by precipitation with Protein A/G PLUS-Agarose (Santa Cruz, sc-2003) for 4 hours at 4 °C. The protein A/G PLUS-Agarose samples were collected, washed, and boiled. Then, the samples were immunoblotted with anti-CTGF or anti-Notch1 antibodies.

### Analysis of bulk RNA sequencing and single-cell RNA sequencing (scRNA-seq) datasets

To assess the associations between gene expression and clinicopathologic parameters, gene expression profiles from the Gene Expression Omnibus (GEO) database were downloaded with the accession numbers GSE14520 [32] and GSE16757 [33]. The TCGA-LIHC dataset was also obtained from the UCSC Xena database. The EPIC algorithm was utilized to measure the abundance of CAFs using the R “IOBR” package [34].

The scRNA-seq data from samples of patients with early-relapsed and primary HCC were obtained from the China National GeneBank DataBase with accession number CNP0000650 (https://db.cngb.org/search/project/CNP0000650/) [35]. The data were normalized and scaled with the SCTransform function in the R Seurat package (v5.0.1). The principal component analysis (PCA) was performed to reduce the dimensionality, and the T-distributed Stochastic Neighbor Embedding (t-SNE) was applied for non-linear dimensional reduction. The CCAIntegration method was used to remove batch effects across different samples. Cell clusters were annotated according to known markers [35]. The enrichment scores of the Notch signaling pathway were calculated using the “AddModuleScore” function in Seurat.

### Functional enrichment analysis

Gene sets of molecular pathways were obtained from the Molecular Signatures Database (MSigDB). The Gene Set Enrichment Analysis (GSEA) analysis was conducted using the R “clusterProfiler” package [36].

### Molecular docking analysis

Protein structures for NOTCH1 (UniProt ID: P46531) and CTGF (UniProt ID: P29279) were obtained from the AlphaFold Protein Structure Database (https://alphafold.ebi.ac.uk/). Protein-protein docking was performed using HDOCK SERVER (http://hdock.phys.hust.edu.cn/), a hierarchical protein-protein docking approach based on a hybrid algorithm of template-based modeling and ab initio free docking [37]. The binding sites between the proteins are shown in **Table S4-1**. HawkDOCK SERVER (http://cadd.zju.edu.cn/hawkdock/) employing the MM/GBSA (Molecular Mechanics/Generalized Born Surface Area) method was used to calculate protein-protein binding free energies and rank amino acid residue contributions to binding (Table S4-2) [38]. The docking Score was used as the evaluation criterion for the docking results. The optimal docking model was selected from the top 10 conformations generated by the docking simulation, and the binding energy was calculated. Finally, the interacting residues with the highest binding energy rankings were visualized using PyMOL 2.4 software, providing a clear illustration of the protein-protein interaction interface.

### Statistical methods

The R (v4.2.2) and GraphPad Prism (v10.2) software were used to conduct the statistical analysis. The Student’s *t*-test was used to compare continuous variables. For survival analysis, the optimal cutoff point for continuous variables was set using the R package “survminer”. Overall survival (OS) and recurrence-free survival (RFS) were examined via Kaplan-Meier survival curves and the log-rank test. Two-sided P-values less than 0.05 were considered significant.

## Results

### High CAF abundance was correlated with the poor prognosis in patients with HCC

**Fig. 1:**
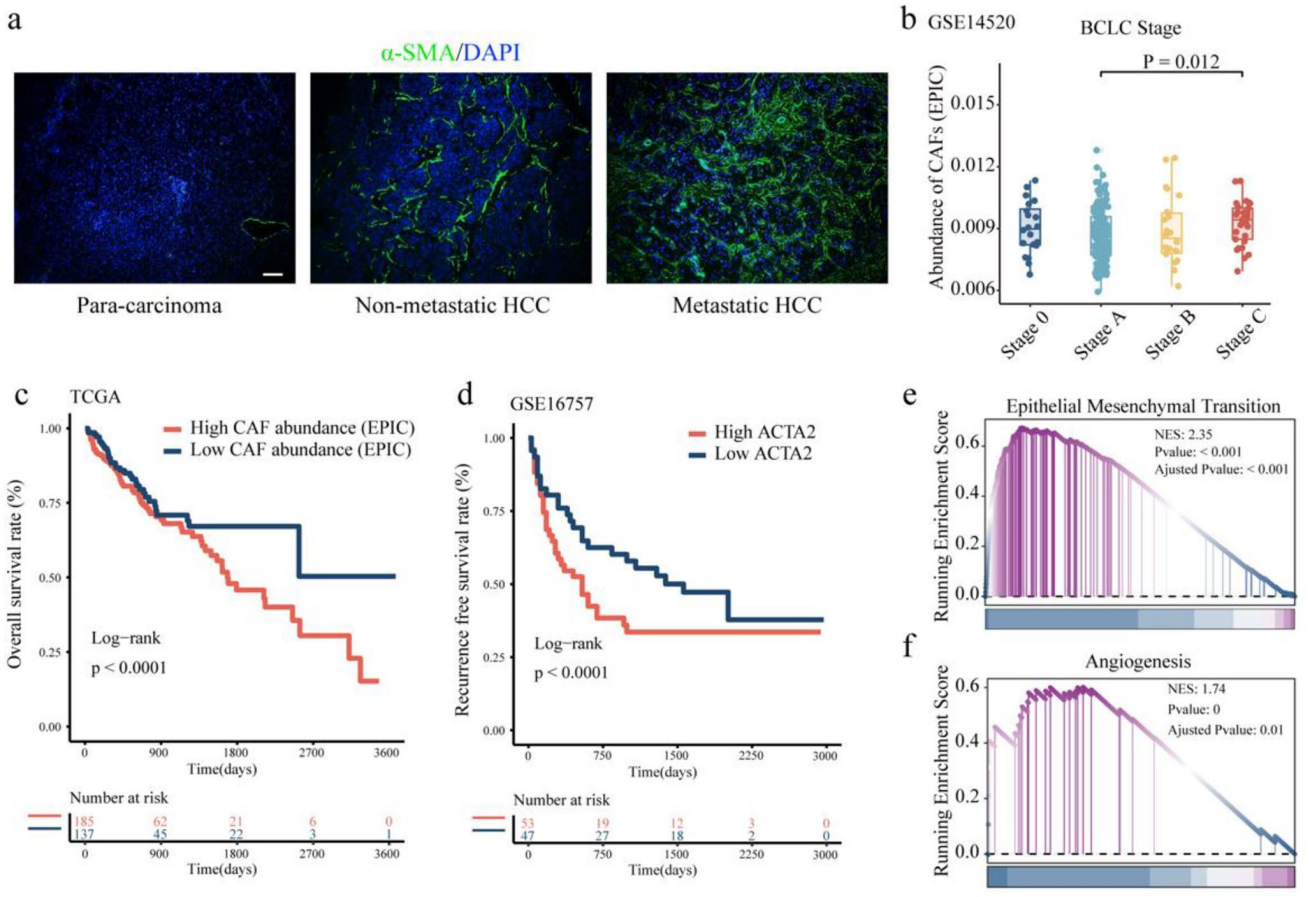
High CAF abundance was associated with poor prognosis in HCC **a.** The protein expression of α-SMA in para-carcinoma tissues, HCC tissues from patients without metastasis, and HCC tissues from patients with metastasis were examined by IF staining (Scale bar, 50 μm). **b.** The abundance of CAFs in clinical samples of BCLC stages of HCC. **c.** OS probabilities of HCC patients were negatively correlated with high CAF abundance. **d.** RFS probabilities of HCC patients were negatively correlated with high ACTA2 expression. **e-f.** GSEA analysis showed enrichment in EMT (**e**) and angiogenesis (**f**) and in the high-CAF abundance group

The expression of the CAF marker α-SMA was examined via IF staining in adjacent normal liver tissues, nonmetastatic HCC tissues, and metastatic HCC tissues. Increased α-SMA was detected in HCC tissue compared with normal paracarcinoma tissue, and the expression of α-SMA was greater in HCC tissues from patients with metastasis than in HCC tissues from patients without metastasis (Fig. 1a). Using cohorts from the Gene Expression Omnibus (GEO) database and The Cancer Genome Atlas (TCGA) database, we assessed the abundance of CAFs in HCC tissues via the EPIC algorithm [34] and analyzed their correlation with clinicopathological features. Data derived from the GSE14520 dataset revealed that CAF abundance was significantly greater in HCC tissues from advanced stages than in those from earlier stages (Fig. 1b). In addition, high CAF abundance was found to be significantly correlated with reduced OS of HCC patients (Fig. 1c). Data from the GSE16757 dataset revealed that HCC patients with high expression levels of ACTA2 had inferior RFS than those with low ACTA2 expression (Fig. 1d).

Furthermore, we classified different HCC tissue samples in the cohort from the TCGA database as having high CAF abundance or low CAF abundance on the basis of the median value. By GSEA, we discovered a significant enrichment of genes related to epithelial‒mesenchymal transition (EMT) and angiogenesis in the high-CAF-abundance samples (Fig. 1e-f), reinforcing our hypothesis that CAFs are mediators of HCC progression.

### CAFs promoted HCC cell proliferation, migration, and invasion via EVs

To explore the role of CAFs in HCC progression, primary NFs and CAFs were isolated from HCC tissues and corresponding adjacent normal tissues. Then, the cell morphologies were observed via a light microscope. CAFs and NFs both displayed typical spindle-shaped morphologies (Fig. S1a). In addition, α-SMA and FAP levels were much higher in CAFs than in NFs, which was verified by IF staining (Fig. S1b) and western blotting (Fig. S1c). Together, CAFs and NFs were isolated successfully.

**Fig. 2:**
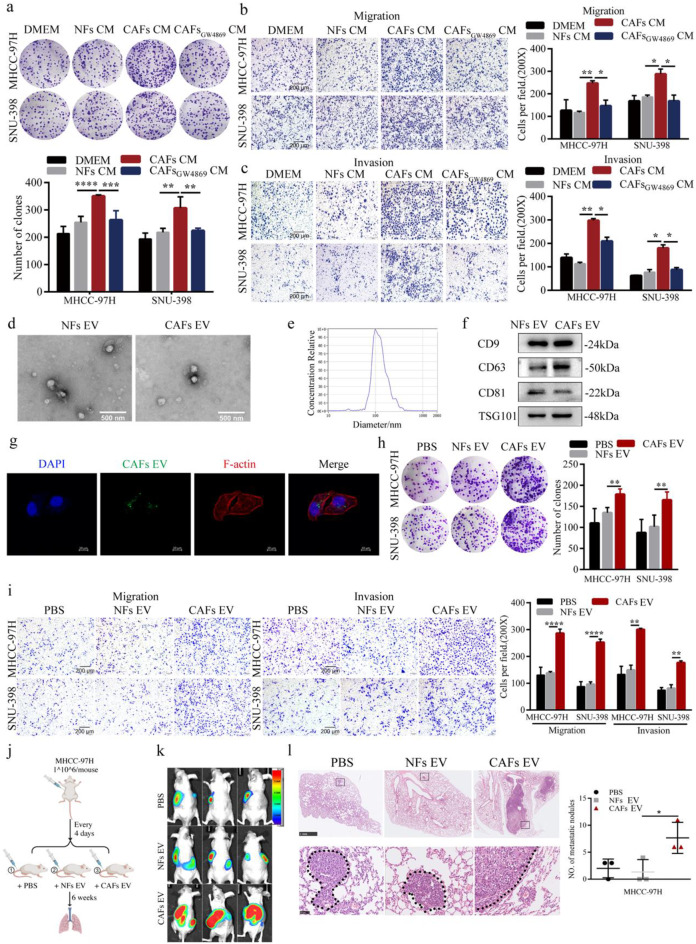
CAF-derived EVs promoted the proliferation, migration, and invasion of HCC cells in vitro and in vivo **a.** HCC cell proliferation was assessed by colony formation assay after 24 h pretreatment with NFs-CM, CAFs-CM, and CAFs-CM in the presence of GW4869. **b-c.** HCC cell migration (**b**) and invasion (**c**) were assessed by Transwell assay after 24 h pretreatment with NFs-CM, CAFs-CM, and CAFs-CM in the presence of GW4869 (Scale bar, 100 μm). **d.** Transmission electron microscope images of CAFs-EV and NFs-EV. **e.** Nanoparticle tracking analysis of CAFs-EV. **f.** Western blot analysis of EV markers in CAFs-EV and NFs-EV. **g.** Uptake of PKH67-labeled CAFs-EV by MHCC-97 H cells observed via confocal microscopy (Scale bar, 10 μm). **h.** HCC cell proliferation was assessed by colony formation assay after 24 h pretreatment with CAFs-EV and NFs-EV. **i.** HCC cell migration and invasion were assessed by Transwell assay after 24 h pretreatment with CAFs-EV and NFs-EV. **j.** Schematic of the experimental process. **k.** Bioluminescence images of orthotopically transplanted HCC tumors in different groups. **l.** H&E staining of tumor lung metastases in different groups (Top scale bar, 1 mm; bottom scale bar, 100 μm)

To elucidate the effect of CAFs on HCC cells, CAF-CM and NF-CM were collected to treat HCC cells. A colony formation assay revealed that, compared with NF-CM or DMEM, CAF-CM promoted the proliferative ability of HCC cells (Fig. 2a). Furthermore, the Transwell assays were performed to test the migratory and invasive abilities of HCC cells. As shown in Fig. 2b-c, compared with NF-CM or DMEM, CAF-CM significantly increased HCC cell migration and invasion.

Previous findings have revealed that CAFs can promote tumor progression by secreting EVs [21]. GW4869, an EV secretion inhibitor, was used to inhibit EV production in CAFs, after which the CM was collected. GW4869 treatment suppressed the pro-proliferative, pro-migratory, and pro-invasive effects of CAF-CM on HCC cells (Fig. 2a-c), suggesting that EVs are critical effectors in CAF-CM that exert those effects.

EVs from CAF-CM (CAF-EVs) and NF-CM (NF-EVs) were isolated and characterized via TEM, NTA, and western blotting (Fig. 2d-f, Fig. S1d). CAF-EVs and NF-EVs labeled with PKH67 were internalized by MHCC-97 H and SNU-398 cells, as shown by their green fluorescence (Fig. 2g, Fig. S1e). Compared with those treated with NF-EVs or PBS, HCC cells treated with CAF-EVs presented significantly greater capabilities of colony formation, migration, and invasion (Fig. 2h-i).

The roles of CAF-derived EVs in educating HCC cells were further evaluated *in vivo* (Fig. 2j). Compared with those treated with NF-EVs or PBS, the orthotopic liver xenograft model treated with CAF-EVs presented increased luminescence intensity and number of lung metastatic nodules (Fig. 2k-l). Collectively, these results indicate that CAF-derived EVs enhance HCC cell proliferation, migration, and metastasis.

### CTGF was upregulated in CAF-derived EVs and promoted the migration and invasion of HCC cells *in vitro*

**Fig. 3:**
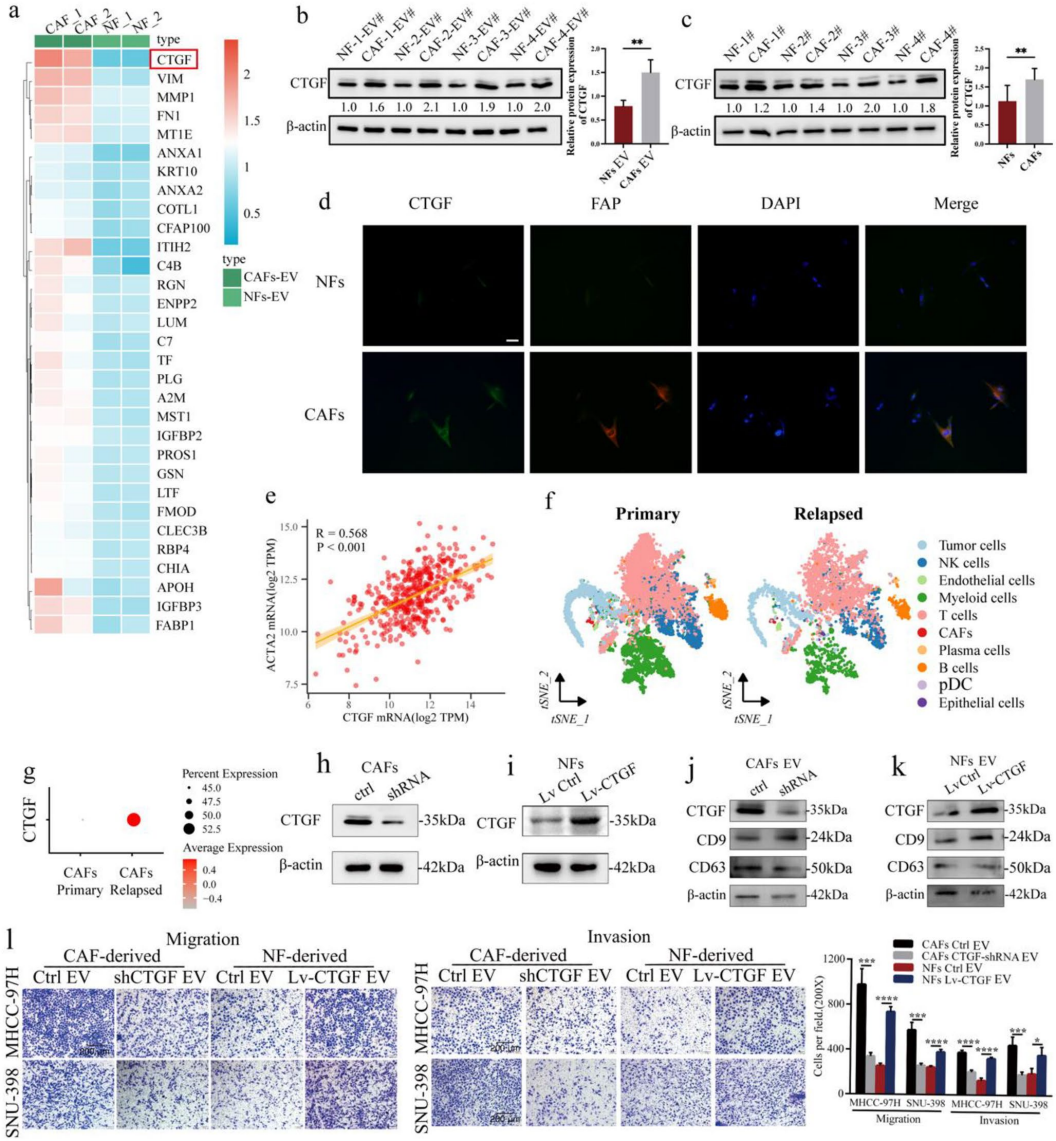
CTGF was enriched in CAF-derived EVs and promoted the migration and invasion of HCC cells in vitro **a.** Heat map of upregulated proteins in CAFs-EV compared to NFs-EV. **b**. The expression of CTGF in EVs from paired NFs and CAFs was assessed by western blot. **c.** The expression of CTGF in paired NFs and CAFs was assessed by western blot. **d.** The expression of CTGF in NFs and CAFs was assessed by IF staining (Scale bar, 20 μm). **e.** The correlation between CTGF expression and ACTA2 expression based on the cohort from the TCGA database. **f.** t-SNE plot of cells in the TME of human primary and early-relapsed HCC. **g.** CTGF expression in CAFs from human primary and early-relapsed HCC. **h**. The knockdown efficiency of CTGF in CAFs was validated by western blot. **i.** The overexpression efficiency of CTGF in NFs was validated by western blot. **j.** The expression of CTGF in EVs from indicated CAFs was measured by western blot. **k.** The expression of CTGF in EVs from indicated NFs was measured by western blot. **l.** HCC cell migration and invasion were assessed by Transwell assay after 24 h pretreatment with EVs from indicated cells (Scale bar, 100 μm)

Proteins are frequently encapsulated in EVs and are implicated in intercellular communication [39]. To identify proteins that are transferred to HCC cells by CAF-EVs, we conducted iTRAQ proteomic analysis of CAF-EVs and NF-EVs. Data analyses revealed that 31 proteins were upregulated in CAF-EVs (Fig. 3a), and 23 proteins were elevated in NF-EVs (Fig. S1f). Among the 54 differentially expressed proteins, connective tissue growth factor (CTGF) was more highly increased (more than 3-fold) in the CAF-EVs than in the NF-EVs (Fig. 3a). These findings were further validated by western blotting (Fig. 3b). We also detected CTGF levels in isolated CAFs and NFs. As confirmed by western blotting (Fig. 3c) and IF staining (Fig. 3d), CTGF expression was increased in CAFs compared to NFs. Furthermore, based on RNA-seq data from the TCGA database, we detected a positive association between CTGF expression and ACTA2 expression in HCC samples (Fig. 3e). To determine the expression pattern of CTGF in CAFs, we analyzed a published scRNA-seq dataset of HCC samples and revealed that CTGF expression was elevated in CAFs from early-relapsed HCC tissues compared with that in CAFs from primary HCC tissues (Fig. 3f-g) [35]. In addition, we also found that compared with tumor cells, the expression levels of CTGF were higher in CAFs (Fig. S1g-h). Taken together, these findings suggested that CTGF is upregulated in CAF-derived EVs.

To further investigate the role of CTGF in the tumor-promoting effects of CAF-derived EVs, the expression of CTGF was downregulated in CAFs and upregulated in NFs (Fig. 3h-i). As confirmed by western blotting, CTGF expression was also downregulated in CAF-EVs and upregulated in NF-EVs (Fig. 3j-k). Transwell assays revealed that CTGF downregulation in CAF-EVs significantly inhibited the migration and invasion of HCC cells, whereas CTGF overexpression in NF-EVs promoted these abilities in HCC cells (Fig. 3l). These findings suggest that CAF-EVs promote HCC cell migration and invasion through CTGF.

### High CTGF expression was closely associated with aggressive clinicopathological features in HCC patients

**Fig. 4:**
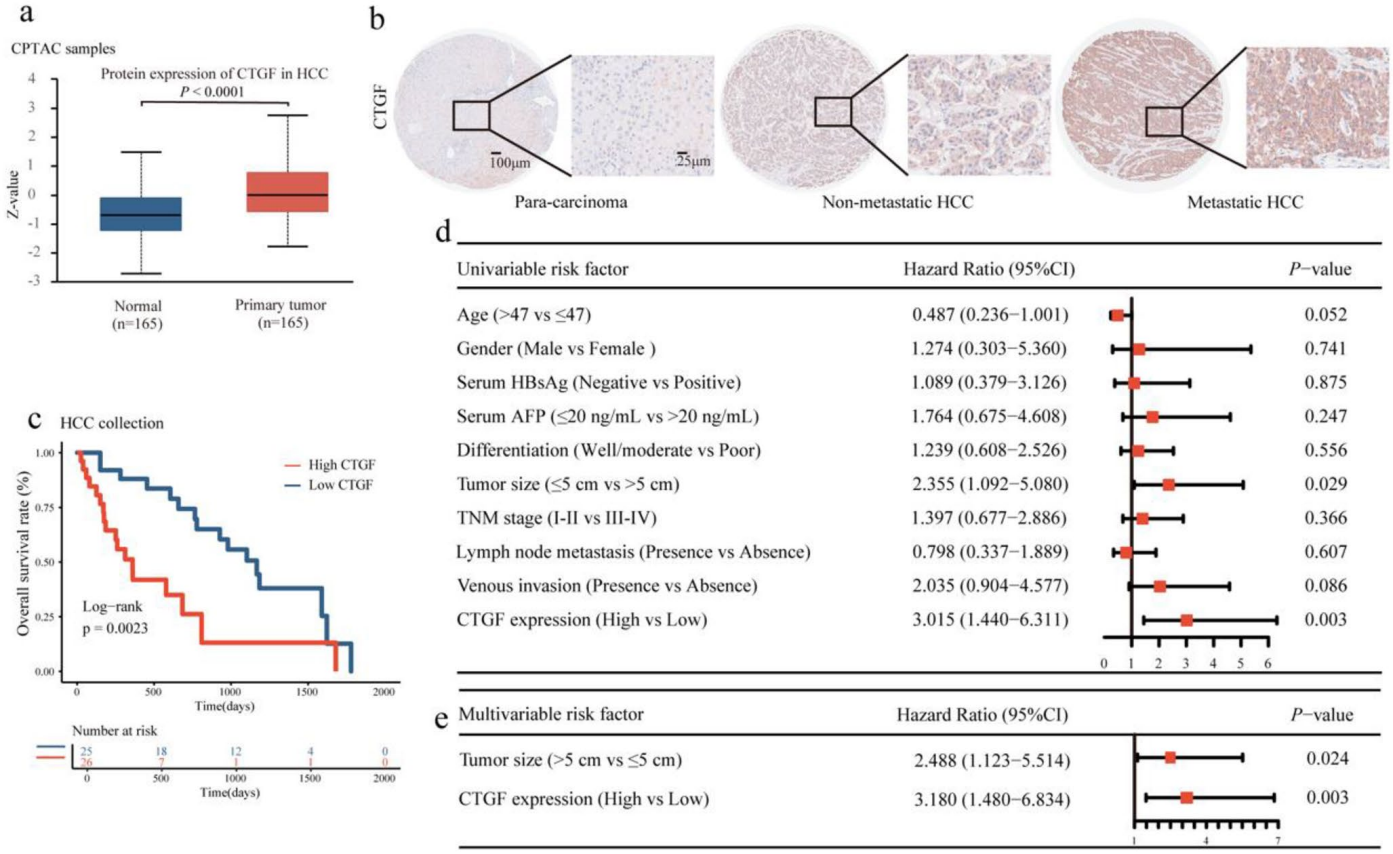
CTGF was closely associated with clinicopathological features in HCC patients **a.** The protein expression of CTGF between normal liver tissues and primary HCC tissues based on the cohort from the CPTAC database. **b.** The protein expression of CTGF in para-carcinoma tissues, HCC tissues from patients without metastasis, and HCC tissues from patients with metastasis were examined by IHC staining (Left scale bar, 100 μm; right scale bar, 25 μm). **c.** High CTGF expression was associated with decreased OS of HCC patients. **d-e.** Forest plot showing the association between CTGF expression and HCC survival using univariate **(d)** and multivariate **(e)** analyses. (CI, confidence interval)

We further investigated the clinical relevance of CTGF expression. Based on data from the Clinical Proteomic Tumor Analysis Consortium (CPTAC) database, CTGF expression in primary tumor tissues was significantly upregulated compared with that in adjacent normal tissue in HCC patients (Fig. 4a). Similar results were observed in paired normal paracarcinoma tissue and HCC tissue, and CTGF expression was upregulated in the HCC tissue of patients with metastasis compared with the HCC tissue of patients without metastasis (Fig. 4b).

To explore the clinical significance of CTGF in HCC, we performed IHC staining analysis on 51 HCC tissues and analyzed the associations between CTGF expression and the clinicopathologic features of HCC patients. We found that CTGF expression was significantly correlated with tumor-node-metastasis (TNM) stage, while there was no significant correlation between CTGF expression and age, sex, serum HBsAg, serum AFP, differentiation grade, tumor size, lymphatic metastasis, or venous invasion (Table 1).

**Table 1:** Association of CTGF expression with clinicopathologic features in 51 HCC cases

Features	Total (n = 51)	Low CTGF (n = 25)	High CTGF (n = 26)	*P*-Value
Age, years				0.490
≤ 47 (median)	28 (54.9%)	12 (48.0%)	16 (61.5%)	
> 47	23 (45.1%)	13 (52.0%)	10 (38.5%)	
Gender				0.350
Male	47 (92.2%)	22 (88.0%)	25 (96.2%)	
Female	4 (7.8%)	3 (12.0%)	1 (3.8%)	
Overall survival, median, months	41.5 months	81.6 months	21.7 months	**0.002**
Serum HBsAg				0.465
Negative	8 (15.7%)	5 (20.0%)	3 (11.5%)	
Positive	43 (84.3%)	20 (80.0%)	23 (88.5%)	
Serum AFP (ng/mL)				0.683
Low (≤ 20)	12 (23.5%)	7 (28.0%)	5 (19.2%)	
High (> 20)	39 (76.5%)	18 (72.0%)	21 (80.8%)	
Differentiation				0.872
Well/moderate	29 (56.9%)	15 (60.0%)	14 (53.8%)	
Poor	22 (43.1%)	10 (40.0%)	12 (46.2%)	
Tumor size				0.205
Small (≤ 5 cm)	19 (37.3%)	12 (48.0%)	7 (26.9%)	
Large (> 5 cm)	32 (62.7%)	13 (52.0%)	19 (73.1%)	
TNM stage (AJCC)				**0.034**
I–II	35 (68.6%)	21 (84.0%)	14 (53.9%)	
III–IV	16 (31.4%)	4 (16.0%)	12 (46.1%)	
Lymph node metastasis				0.173
Absence	40 (78.4%)	22 (88.0%)	18 (69.2%)	
Presence	11 (21.6%)	3 (12.0%)	8 (30.8%)	
Venous invasion				0.140
Absence	42 (82.4%)	23 (92.0%)	19 (73.1%)	
Presence	9 (17.6%)	2 (8.0%)	7 (26.9%)	

Note. Statistical significance (P < 0.05) is shown in bold. Chi-square test and Fisher’s exact test

Additionally, the association between CTGF expression and clinical survival was assessed via Kaplan‒Meier curves and the log-rank test. High CTGF expression was strongly correlated with decreased OS in patients with HCC (Fig. 4c). Univariate and multivariate Cox regression analyses further confirmed CTGF expression as an independent predictor of the survival of HCC patients in addition to tumor size (Fig. 4d-e). Collectively, these data indicate that high CTGF expression is significantly associated with poor clinical outcomes in HCC patients.

### CTGF derived from CAF-EVs activates Notch1 signaling in HCC cells

Numerous studies have highlighted the role of EVs in signal transduction [40]. However, the underlying pathways involved in CAF-EVs-mediated promoting roles in HCC proliferation and invasion remain unknown. The Notch signaling pathway has been highly conserved throughout evolution and plays a pivotal role in tumorigenesis and tumor progression [41]. Our previous study indicated that CAFs promote tumor stemness by activating the Notch signaling pathway [6]. Herein, we investigated whether CTGF transferred by CAF-EVs activated downstream Notch signaling in HCC cells, thereby promoting tumor metastasis. We classified different HCC tissue samples from the TCGA-LIHC cohort as CTGF^high^ or CTGF^low^ based on median expression. The GSEA revealed significant enrichment of the gene sets for the Notch pathway in the CTGF^high^ group (Fig. S2a). Furthermore, gene correlation analysis of the TCGA-LIHC cohort revealed positive correlations between CTGF expression and the expression of Notch pathway genes (Fig. S2b-d). In addition, CTGF expression was found to be positively correlated with the expression of Snail1 (Fig. S2e), a key effector of EMT [42].

**Fig. 5:**
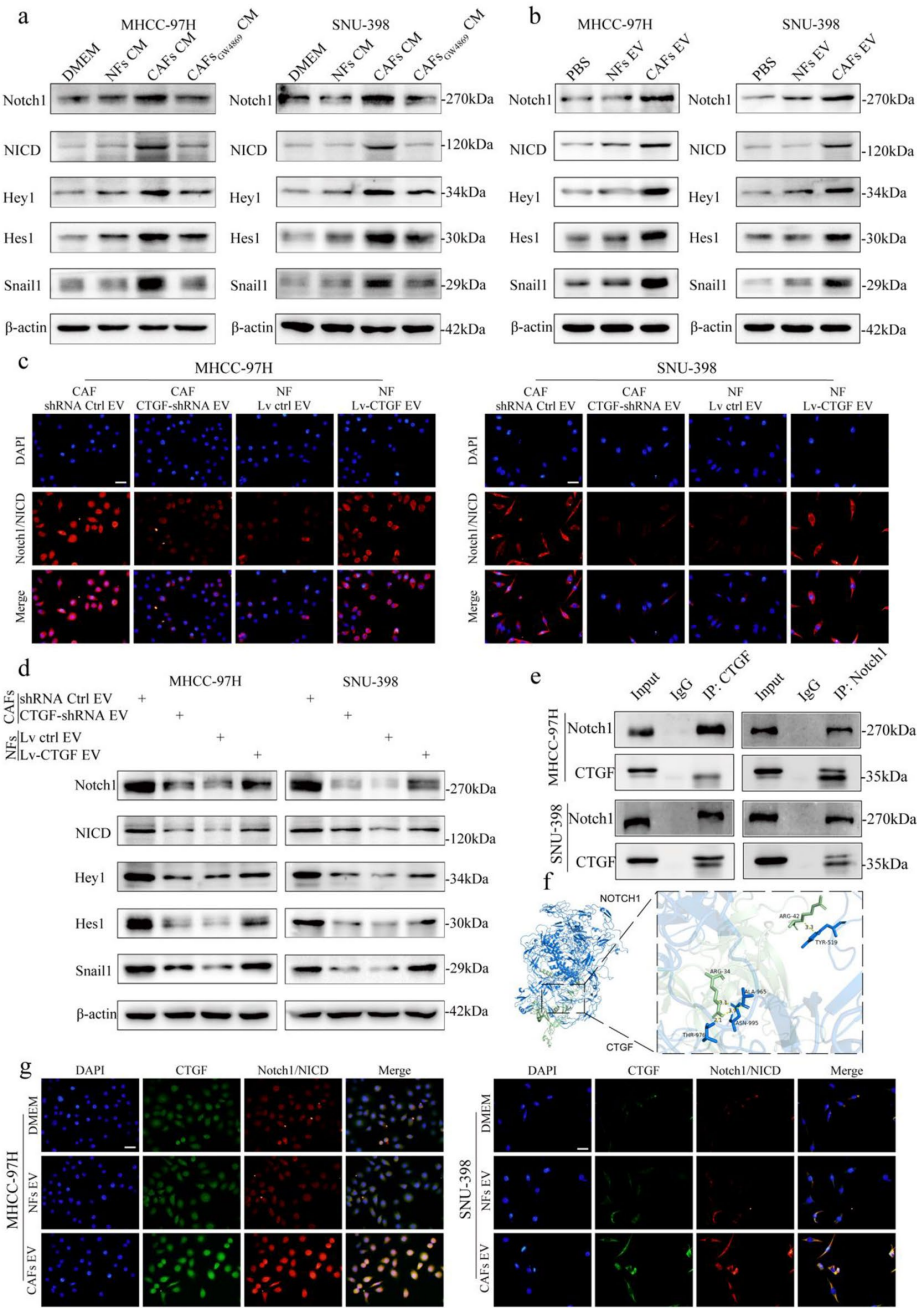
CTGF derived from CAF-EVs activates Notch1 signaling in HCC cells **a.** Expression of Notch1 pathway and Snail1 proteins in HCC cells after 24 h pretreatment with NFs-CM, CAFs-CM, and CAFs-CM in the presence of GW4869. **b.** Expression of Notch1 pathway and Snail1 proteins in HCC cells after 24 h pretreatment with NFs-EV and CAFs-EV. **c.** IF images of MHCC-97 H and SNU-398 cells treated with EVs from indicated cells to assess the expression of Notch1/NICD (red) (Scale bars, 20 μm). **d.** Expression of Notch1 pathway and Snail1 proteins in HCC cells after 24 h pretreatment with EVs from indicated cells. **e.** Reciprocal co-IP experiments with anti-Notch1 or anti-CTGF antibodies in HCC cells. **f.** The 3D molecular docking results showed that the conformations of CTGF and Notch1 receptor contain good binding interactions (binding free energy = − 136.93 kcal/mol, HDOCK Docking Score = − 319.72). **g.** IF images of MHCC-97 H and SNU-398 cells treated with PBS, NF-derived EVs, and CAF-derived EVs showed colocalization of CTGF (green) Notch1/NICD (red) (Scale bars, 20 μm)

By western blot, we found that, compared with NF-CM or DMEM, treatment with CAF-CM increased the expression levels of Notch1, NICD, HES1, HEY1, and Snail1 in HCC cells, whereas GW4869 treatment inhibited the increases in expression of the Notch1 pathway proteins (Fig. 5a). In addition, compared with PBS or NF-EVs, treatment with CAF-EVs increased the expression levels of Notch1, NICD, HES1, HEY1, and Snail1 in HCC cells (Fig. 5b). IF staining revealed that compared with those in the control groups, HCC cells treated with CTGF-downregulated CAF-EVs presented decreased Notch1/NICD expression (red), and HCC cells treated with CTGF-overexpressing NF-EVs presented increased Notch1/NICD expression (red) (Fig. 5c). Furthermore, western blot assays revealed that CTGF downregulation in CAF-EVs inhibited the expression of Notch pathway proteins and Snail1, whereas upregulation of CTGF in NF-EVs increased their expression levels (Fig. 5d). Co-IP experiments demonstrated a physical interaction between CTGF and Notch1 (Fig. 5e). Molecular docking was further employed to investigate interactions between CTGF and Notch1. The binding free energy lower than − 5.0 kcal/mol indicates that the conformations interact well. In this study, molecular docking results showed that the conformations of CTGF and Notch1 receptor contain good binding interactions (Fig. 5f), and the interactions were also reliable (binding free energy = − 136.93 kcal/mol, HDOCK Docking Score = − 319.72). IF staining also revealed the colocalization of CTGF (green) and Notch1 (red) in HCC cells treated with CAF-derived EVs (Fig. 5g). These findings suggested that CTGF derived from CAF-EVs activated the Notch1 signaling pathway in HCC cells.

### CTGF promoted HCC metastasis via the Notch1/Snail1 signaling

We then aimed to investigate whether the tumor-promoting effects of CTGF are mediated through the Notch1 signaling pathway. CTGF and Notch1 are sequentially regulated in CAFs and HCC cells, respectively. The downregulation and overexpression efficiency of Notch1 in HCC cells was confirmed by western blotting (Fig. S3a-b).

**Fig. 6:**
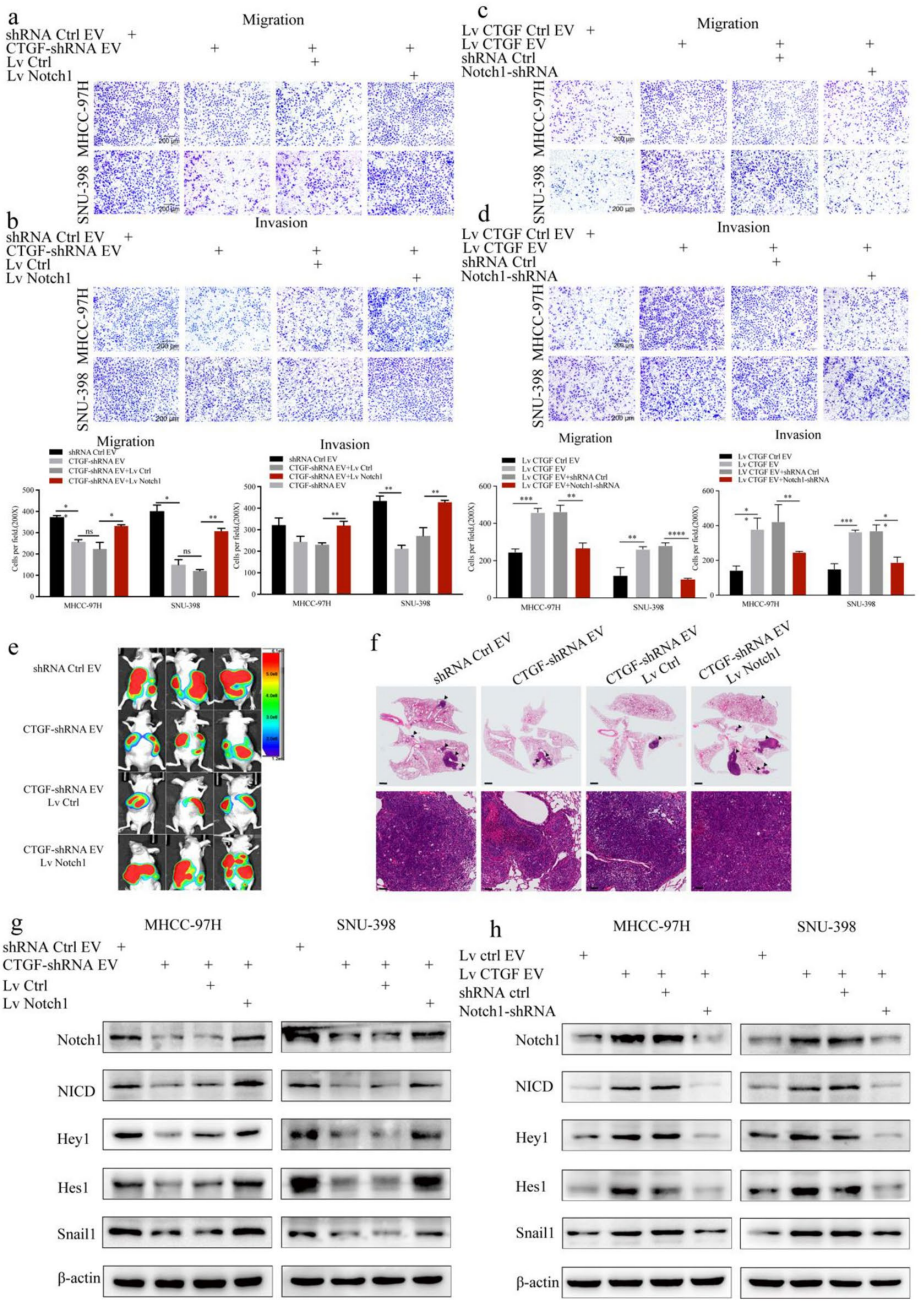
CTGF derived from CAF-EVs promoted HCC cell metastasis by activating the Notch1 signaling pathway a**-b.** Changes in migration (**a**) and invasion (**b**) capacity in Notch1-overexpressing HCC cells treated with EVs from CTGF-downregulated CAFs were measured by Transwell assays. **c-d.** Changes in migration (**c**) and invasion (**d**) capacity in Notch1-silenced HCC cells treated with EVs from CTGF-upregulated NFs were measured by Transwell assays. **e.** Bioluminescence images of orthotopically transplanted HCC tumors in different groups. **f.** H&E staining of tumor lung metastases in different groups (Top scale bar, 1 mm; bottom scale bar, 100 μm). **g.** Changes in the Notch1 pathway and Snail1 protein expression in Notch1-overexpressing HCC cells treated with EVs from CTGF-downregulated CAFs were measured by western blot. **h.** Changes in the Notch1 pathway and Snail1 protein expression in Notch1-silenced HCC cells treated with EVs from CTGF-upregulated NFs were measured by western blot

Transwell assays revealed that CTGF downregulation in CAF-EVs inhibited the migratory and invasive capabilities of HCC cells, whereas sequential upregulation of Notch1 in HCC cells restored these capacities (Fig. 6a-b). Moreover, CTGF upregulation in NF-EVs promoted the migratory and invasive capabilities of HCC cells, whereas subsequent Notch1 downregulation in HCC cells inhibited these capacities (Fig. 6c-d). Furthermore, the luminescence intensity and number of lung metastatic nodules were lower in the orthotopic liver xenograft model treated with EVs from CTGF-downregulated CAFs than in the control group (Fig. 6e-f). Conversely, sequential upregulation of Notch1 in HCC cells increased the luminescence intensity and the number of metastatic nodules in lung tissues (Fig. 6e-f).

In addition, by western blot, we found that CTGF downregulation in CAF-EVs inhibited the expression of the Notch pathway proteins and Snail1, whereas sequential upregulation of Notch1 in HCC cells restored their expression levels (Fig. 6g). Conversely, CTGF upregulation in NF-EVs promoted the expression of the Notch pathway proteins and Snail1, whereas sequential downregulation of Notch1 in HCC cells reversed their expressions (Fig. 6h). Collectively, these results indicate that CTGF derived from CAF-EVs facilitates HCC cell proliferation and metastasis through the Notch1/Snail1 signaling axis.

### The Notch1/Snail1 axis was associated with poor prognosis in HCC

**Fig. 7:**
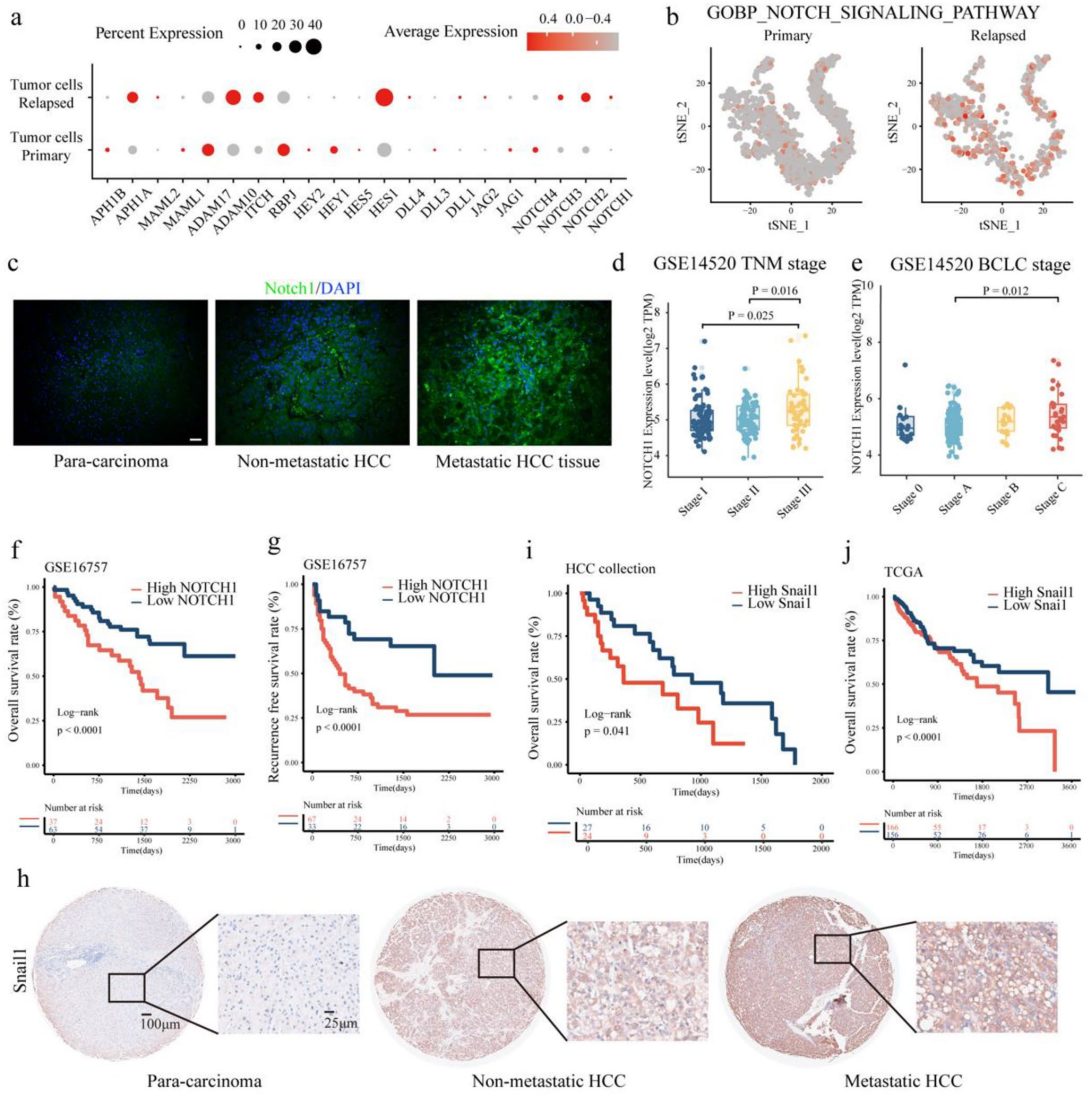
The Notch1/Snail1 signaling axis was associated with poor prognosis in HCC **a.** Dot plot showing the expression levels of the Notch pathway genes in tumor cells from human primary and early-relapsed HCC. **b.** Gene module scores of the Notch signaling pathway in tumor cells from human primary and early-relapsed HCC. **c**. The protein expression of CTGF in para-carcinoma tissues, HCC tissues from patients without metastasis, and HCC tissues from patients with metastasis were examined by IF staining (Scale bar, 20 μm). **d-e.** Notch1 mRNA expression in HCC samples of different TNM stages (**d**) and BCLC stages (**e**). **f-g.** High Notch1 expression was negatively correlated with decreased OS (**f**) and RFS (**g**) probabilities in HCC patients. **h.** The protein expression of Snail1 in para-carcinoma tissues, HCC tissues from patients without metastasis, and HCC tissues from patients with metastasis were examined by IHC staining (Left scale bar, 100 μm; right scale bar, 25 μm). **i.** High Snail1 expression was negatively associated with decreased OS probabilities in HCC patients. **j.** High Snail1 expression was negatively associated with decreased OS probabilities in HCC patients from the TCGA cohort

The scRNA-seq data were then re-analyzed to explore the involvement of the Notch pathway in HCC progression [35]. We found that the expression levels of Notch pathway proteins were higher in relapsed HCC tumor cells than in primary HCC cells (Fig. 7a). Moreover, we examined the gene module scores of the Notch pathway and found significant enrichment of the Notch pathway in relapsed HCC tumor cells compared with primary HCC cells (Fig. 7b), indicating increased activation of the Notch pathway in relapsed HCC. IF staining revealed increased Notch1 expression in HCC tissue compared with adjacent normal liver tissue, and the expression of Notch1 was greater in HCC tissues from patients with metastasis than in HCC tissues from patients without metastasis (Fig. 7c). Analysis of RNA-seq data from the GSE14520 dataset revealed a significant correlation between high Notch1 expression and advanced TNM stages and BCLC stages (Fig. 7d-e). Survival analysis of the GSE16757 dataset revealed that high Notch1 expression was strongly associated with decreased OS and RFS in patients with HCC (Fig. 7f-g).

**Fig. 8:**
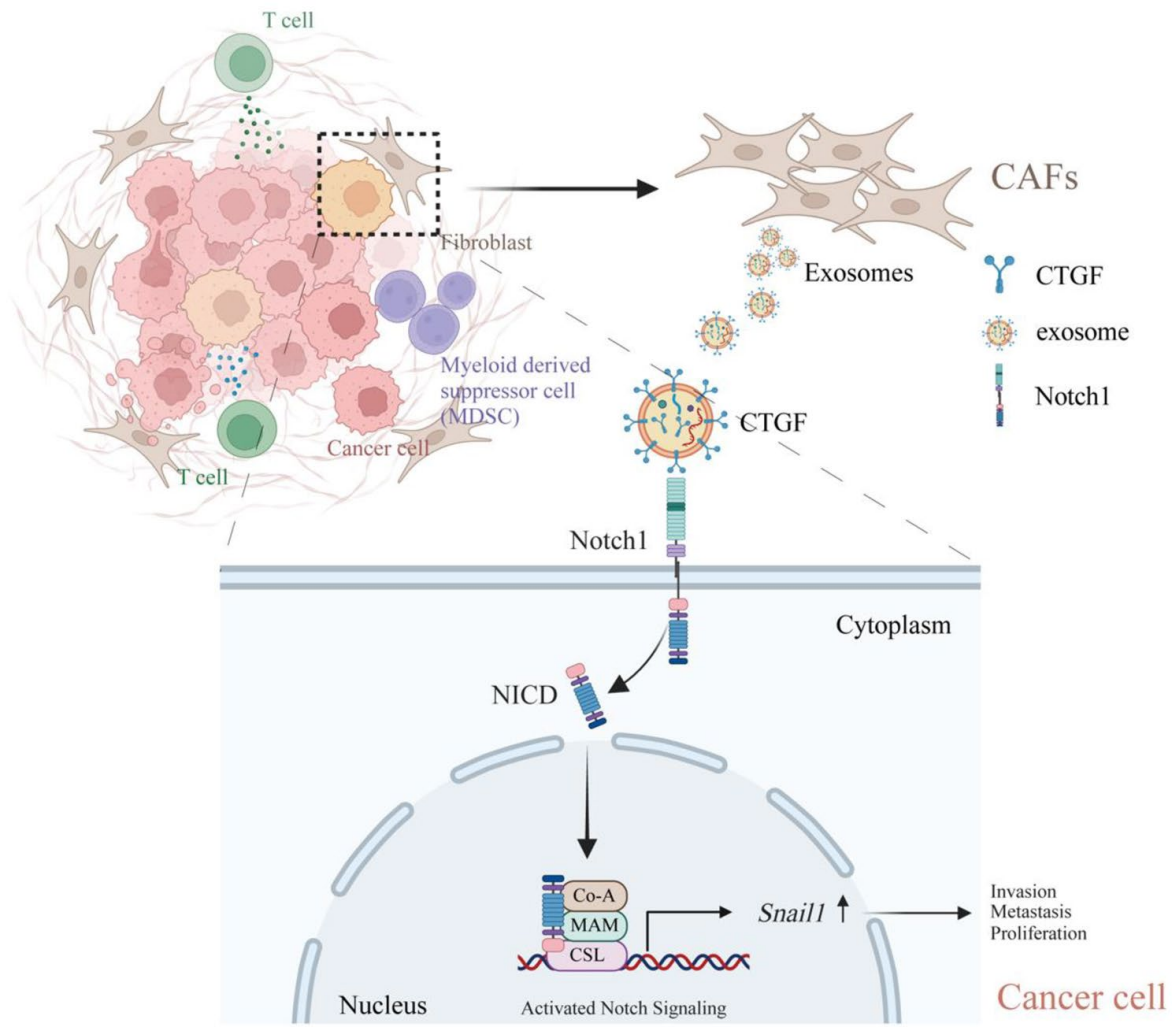
CTGF derived from CAF-EVs promoted HCC cell proliferation and metastasis by activating the Notch1/Snail1 signaling pathway The workflow was created with BioRender (https://www.biorender.com/)

Furthermore, we detected an increase in Snail1 expression in HCC tissue from patients with metastasis compared with HCC tissue from patients without metastasis (Fig. 7h). IHC staining of Snail1 in 51 HCC tissues was performed to explore the correlation between Snail1 expression and the clinicopathological characteristics of HCC patients. Snail1 expression was found to be significantly correlated with tumor size, while no significant associations were observed between Snail1 expression and age, sex, serum HBsAg, serum AFP, differentiation grade, TNM stage, lymphatic metastasis, or venous invasion (Table 2). Furthermore, high Snail1 expression was strongly associated with decreased OS in patients with HCC (Fig. 7i). The survival analysis of the TCGA-LIHC cohort revealed the same trend (Fig. 7j). Taken together, these findings suggest that the Notch1/Snail1 axis is associated with poor clinical outcomes in HCC patients. In conclusion, CTGF derived from CAF-EVs activated the Noth1/Snail1 signaling axis to promote HCC metastasis by interacting with the Notch1 receptor (Fig. 8).

**Table 2:** Association of Snail1 expression with clinicopathologic features in 51 HCC cases

Features	Total (n = 51)	Low Snail1 (n = 27)	High Snail1 (n = 24)	*P*-Value
Age, years				> 0.999
≤ 47 (median)	28 (54.9%)	15 (55.6%)	13 (54.2%)	
> 47	23 (45.1%)	12 (44.4%)	11 (45.8%)	
Gender				0.612
Male	47 (92.2%)	24 (88.9%)	23 (95.8%)	
Female	4 (7.80%)	3 (11.1%)	1 (4.2%)	
Overall survival, median, months	41.5 months	54.8 months	28.0 months	**0.041**
Serum HBsAg				> 0.999
Negative	8 (15.7%)	4 (14.8.0%)	4(16.7%)	
Positive	43 (84.3%)	23 (85.2%)	20 (83.3%)	
Serum AFP (ng/mL)				0.923
Low (≤ 20)	12 (23.5%)	7 (25.9%)	5 (20.8%)	
High (> 20)	39 (76.5%)	20 (74.1%)	19 (79.2%)	
Differentiation				0.629
Well/moderate	29 (56.9%)	14 (51.8%)	15 (62.5%)	
Poor	22 (43.1%)	13 (48.2%)	9 (37.5%)	
Tumor size				**0.046**
Small (≤ 5 cm)	19 (37.3%)	14 (51.8%)	5 (20.8%)	
Large (> 5 cm)	32 (62.7%)	13(48.2%)	19 (79.2%)	
TNM stage (AJCC)				0.534
I–II	35 (68.6%)	17 (63.0%)	18 (75.0%)	
III–IV	16 (31.4%)	10 (37.0%)	6 (25.0%)	
Lymph node metastasis				0.182
Absence	40 (78.4%)	19 (70.4%)	21 (87.5%)	
Presence	11 (21.6%)	8 (29.6%)	3 (12.5%)	
Venous invasion				> 0.999
Absence	42 (82.4%)	22 (81.5%)	20 (83.3%)	
Presence	9 (17.6%)	5 (18.5%)	4 (16.7%)	

Note. Statistical significance (P < 0.05) is shown in bold. Chi-square test and Fisher’s exact test

## Discussion

The poor prognosis of HCC patients is primarily due to high recurrence rates and early metastasis [43]. While early-stage HCC can be treated by surgery, most patients are diagnosed at advanced stages when the cancer is resistant to chemotherapy and targeted therapy [44]. Therefore, it is crucial to explore the molecular mechanisms underlying metastasis and recurrence to identify new therapeutic targets for improving patient outcomes.

The TME is a significant driver of tumor progression, and altering its characteristics or components has shown promise in enhancing cancer treatment efficacy [45, 46]. CAFs, a key component of the TME stroma, can either promote or inhibit tumor progression [47]. CAFs secrete various cytokines and chemokines that influence cells in the TME, contributing to tumor metastasis, angiogenesis, inflammation, and immunosuppression [17]. For example, CAF-secreted CCL5 interacts with CCR3/5 on HCC cells, inhibiting the ubiquitination and degradation of HIF1α and upregulating ZEB1, thereby supporting invasion and metastasis [48]. In our study, we demonstrated that CAFs are essential drivers of HCC progression, with CAF-conditioned medium promoting tumor cell proliferation and migration through yet unclear mechanisms.

In addition to classical paracrine signaling, EV-mediated tumor-stroma interactions play crucial roles in regulating key processes in cancers [19, 49]. EVs can carry and transfer non-coding RNAs, proteins, DNA, and other molecules, modulating intercellular communication in the TME and contributing to tumor progression. For example, EVs from tumor-associated macrophages increase the migratory capacity of gastric cancer cells [50], and CAF-derived EVs facilitate metastasis and chemotherapy resistance in colorectal cancer cells [21]. In this study, we revealed that CTGF is significantly enriched in CAF-derived EVs. CTGF, a member of the cellular communication network (CCN) family, is a multifunctional regulator involved in wound healing and tissue repair [51]. Aberrant expression of CTGF is strongly correlated with ECM deposition and fibrosis, which is consistent with its specific enrichment in CAF-derived EVs [52]. Numerous studies have shown that CTGF is a key driver of tumor progression [53]. In HCC, CTGF secreted by mesenchymal-like HCC cells polarizes macrophages into the M2 state, which reciprocally transfers CCL18 to promote HCC progression [54]. However, limited research has investigated the impact of CTGF from CAF-derived EVs on tumor cells. Our study demonstrated that CTGF is responsible for the tumor-promoting roles of CAF-derived EVs, as shown both *in vitro* and *in vivo*.

Interestingly, several proteins downregulated in CAF-derived EVs have been implicated in the pro-metastatic phenotypes of CAFs. Matrix metalloproteinase 3 (MMP3) has demonstrated metastasis-promoting capabilities across multiple malignancies [55, 56, 57]. In pancreatic cancer, CAF-secreted MMP3 enhances the migratory and invasive capacities of cancer cells *in vitro* [58]. Similarly, in colorectal carcinoma, cancer cell-reprogrammed fibroblasts secrete MMPs and CCL5, thereby facilitating tumor invasion both *in vitro* and *in vivo* [59]. Furthermore, MMP3^+^IL24^+^ myCAFs have been shown to accelerate esophageal squamous cell carcinoma metastasis through ECM degradation and promotion of neovascularization [60]. In addition, despite CAFs being widely recognized for their role in ECM remodeling, our proteomic analysis revealed that multiple ECM-associated proteins (e.g., COL4A1, COL1A1, COL1A2) were significantly downregulated in CAF-derived EVs compared to NF-derived EVs. This result suggests that cargo sorting of EVs may be highly selective and represent an additional regulatory mechanism in CAF-tumor communication. CAFs may preferentially package pro-tumorigenic factors (e.g., CTGF) into EVs while utilizing alternative secretory pathways for ECM-associated proteins. Future studies comparing the complete secretome of CAFs versus the proteome of CAF-derived EVs would provide valuable insights into these differential secretory mechanisms and their biological significance in HCC development.

EVs are known to be key regulators of intercellular signal transduction [61]. However, the mechanism by which CTGF from CAF-derived EVs supports HCC migration and invasion is not well understood. The Notch1 signaling pathway is crucial for regulating tumor metastasis, angiogenesis, immunosuppression, and tumor stemness [62, 63, 64, 65]. Expressions of Notch1 pathway genes are highly upregulated in various cancers and are generally associated with aggressive clinicopathological features [66, 67, 68]. Aberrant activation of Notch1 signaling is also correlated with epithelial‒mesenchymal transition (EMT) and stemness in HCC [69, 70]. Snail1, a key driver of EMT, is associated with poor prognosis and metastasis in many cancers [71, 72, 73]. Our study revealed that Snail1 could be induced by the Notch1 signaling pathway. Clinical correlation analysis revealed that Notch1/Snail1 activity is associated with poor clinical outcomes, including relapse and metastasis, in patients with HCC.

Our study has several limitations that should be acknowledged. First, CAFs exhibit considerable heterogeneity within the TME, with distinct functional subpopulations including myCAFs, iCAFs, and apCAFs. In our study, immunofluorescence characterization revealed that our isolated CAFs predominantly expressed α-SMA, FAP, and vimentin, consistent with a myCAF phenotype. Previous investigations in pancreatic cancer demonstrated that pancreatic stellate cells adopt an iCAF phenotype when treated with a cancer organoid-conditioned medium [15]. However, when these iCAFs are subsequently cultured in monolayer conditions, they rapidly revert to a myofibroblastic state, downregulating inflammatory markers while upregulating α-SMA expression. This phenotypic plasticity suggests that myCAFs represent a more stable phenotype under *in vitro* culture conditions. Future studies should examine the differential protein composition of EVs derived from distinct CAF subpopulations to further elucidate how CAF heterogeneity impacts HCC development and progression. Second, the effects of CTGF derived from CAF-EVs on Notch1 pathway components at the transcriptional or translational level warrant further investigation. Notably, our previous study demonstrated that CAF-derived STC1 activates the Notch1 pathway in HCC cells, promoting NICD nuclear translocation and the formation of transcriptional complexes that bind to the STC1 promoter, establishing an STC1-Notch1 feedforward signal [12]. Studies have shown that Notch1 can induce CTGF mRNA expression in osteoblastic cells [74], while in renal tubular epithelial cells, PLK2-Notch1 interaction activates the Notch pathway and promotes CTGF expression [75]. These findings suggest that Notch1 activation might enhance CTGF transcription in HCC cells, potentially forming a feedforward loop that amplifies pro-metastatic signaling. Future studies using dual-luciferase reporter assays should investigate this regulatory mechanism between CTGF and Notch1 signaling in HCC progression. Third, the effects of CTGF modulation on CAF phenotypes and functions remain to be elucidated. In HCC, cancer cell-derived CTGF promotes CAF proliferation, which subsequently facilitates tumor growth, intravasation, and metastatic dissemination of cancer cells [76]. Similarly, in gastric cancer, cancer cell-derived CTGF enhances the proliferative, invasive, and migratory capabilities of CAFs, associated with increased collagen fiber deposition within the TME [77]. Future investigations should explore whether CTGF can be secreted by HCC cells and modulate CAF functions, potentially establishing a reciprocal signaling loop between cancer cells and tumor stroma that further drives disease progression.

## Conclusions

In summary, our study provides evidence that CAF-derived EVs promote HCC cell metastasis through the Notch1/Snail1 signaling axis. We found that CTGF derived from CAF-EVs can be directly transmitted to HCC cells, increasing their migratory and invasive capacities. CTGF promotes tumor progression by acting on the Notch1 receptor on the tumor cell surface. Thus, targeting the CTGF/Notch1 signaling axis may be a potential strategy to inhibit HCC metastasis.

## Electronic supplementary material

Below is the link to the electronic supplementary material.

Supplementary Material 1

Supplementary Material 2

Supplementary Material 3

## Data Availability

No datasets were generated or analysed during the current study.

## References

[1] A. Villanueva, Hepatocellular carcinoma. N. Engl. J. Med. **380**(15), 1450–1462 (2019) 30970190 10.1056/NEJMra1713263

[2] H. Zhan, X. Zhao, Z. Lu, Y. Yao, X. Zhang, Correlation and survival analysis of distant metastasis site and prognosis in patients with hepatocellular carcinoma. Front. Oncol. **11**, 652768 (2021) 34041022 10.3389/fonc.2021.652768 PMC8141638

[3] V. Catalano, A. Turdo, S. Di Franco, F. Dieli, M. Todaro, G. Stassi, Tumor and its microenvironment: A synergistic interplay. Semin. Cancer Biol. **23**(6 Pt B), 522–32 (2013) 24012661 10.1016/j.semcancer.2013.08.007

[4] T. He, Q. Zhang, P. Xu, W. Tao, F. Lin, R. Liu, et al., Extracellular vesicle-circEHD2 promotes the progression of renal cell carcinoma by activating cancer-associated fibroblasts. Mol. Cancer **22**(1), 117 (2023) 37481520 10.1186/s12943-023-01824-9 PMC10362694

[5] Z. Li, V. Low, V. Luga, J. Sun, E. Earlie, B. Parang, et al., Tumor-produced and aging-associated oncometabolite methylmalonic acid promotes cancer-associated fibroblast activation to drive metastatic progression. Nat. Commun. **13**(1), 6239 (2022) 36266345 10.1038/s41467-022-33862-0 PMC9584945

[6] S. Xiong, R. Wang, Q. Chen, J. Luo, J. Wang, Z. Zhao, et al., Cancer-associated fibroblasts promote stem cell-like properties of hepatocellular carcinoma cells through IL-6/STAT3/notch signaling. Am. J. Cancer Res. **8**(2), 302–316 (2018) 29511600 PMC5835697

[7] B. Chen, Y. Sang, X. Song, D. Zhang, L. Wang, W. Zhao, et al., Exosomal miR-500a-5p derived from cancer-associated fibroblasts promotes breast cancer cell proliferation and metastasis through targeting USP28. Theranostics **11**(8), 3932–3947 (2021) 33664871 10.7150/thno.53412 PMC7914354

[8] Z. Yan, Z. Sheng, Y. Zheng, R. Feng, Q. Xiao, L. Shi, et al., Cancer-associated fibroblast-derived exosomal miR-18b promotes breast cancer invasion and metastasis by regulating TCEAL7. Cell Death Dis. **12**(12), 1120 (2021) 34853307 10.1038/s41419-021-04409-w PMC8636636

[9] X. Jin, Q. Deng, S. Ye, S. Liu, Y. Fu, Y. Liu, et al., Cancer-associated Fibroblast-derived Periostin Promotes Papillary Thyroid Tumor Growth through integrin-FAK-STAT3 Signaling. Theranostics **14**(7), 3014–28 (2024) 10.7150/thno.94207 PMC11103496 38773979

[10] Y. Mori, Y. Okimoto, H. Sakai, Y. Kanda, H. Ohata, D. Shiokawa, et al., Targeting PDGF signaling of cancer-associated fibroblasts blocks feedback activation of HIF-1alpha and tumor progression of clear cell ovarian cancer. Cell. Rep. Med. **5**(5), 101532 (2024) 38670097 10.1016/j.xcrm.2024.101532 PMC11149410

[11] J.W. Eun, J.H. Yoon, H.R. Ahn, S. Kim, Y.B. Kim, S.B. Lim, et al., Cancer-associated fibroblast-derived secreted phosphoprotein 1 contributes to resistance of hepatocellular carcinoma to sorafenib and lenvatinib. Cancer Commun. (Lond.) **43**(4), 455–479 (2023) 36919193 10.1002/cac2.12414 PMC10091107

[12] S. Bai, Y. Zhao, W. Chen, W. Peng, Y. Wang, S. Xiong, et al., The stromal-tumor amplifying STC1-Notch1 feedforward signal promotes the stemness of hepatocellular carcinoma. J. Transl. Med. **21**(1), 236 (2023) 37004088 10.1186/s12967-023-04085-8 PMC10067215

[13] T.X. Huang, X.Y. Tan, H.S. Huang, Y.T. Li, B.L. Liu, K.S. Liu, et al., Targeting cancer-associated fibroblast-secreted WNT2 restores dendritic cell-mediated antitumour immunity. Gut. **71**(2), 333–344 (2022) 33692094 10.1136/gutjnl-2020-322924 PMC8762012

[14] Z.W. Qiu, Y.T. Zhong, Z.M. Lu, N. Yan, R.J. Kong, J.Q. Huang, et al., Breaking physical barrier of fibrotic breast cancer for photodynamic immunotherapy by remodeling tumor extracellular matrix and reprogramming cancer-associated fibroblasts. ACS Nano. **18**(13), 9713–9735 (2024) 38507590 10.1021/acsnano.4c01499

[15] D. Ohlund, A. Handly-Santana, G. Biffi, E. Elyada, A.S. Almeida, M. Ponz-Sarvise, et al., Distinct populations of inflammatory fibroblasts and myofibroblasts in pancreatic cancer. J. Exp. Med. **214**(3), 579–596 (2017) 28232471 10.1084/jem.20162024 PMC5339682

[16] E. Elyada, M. Bolisetty, P. Laise, W.F. Flynn, E.T. Courtois, R.A. Burkhart, et al., Cross-species single-cell analysis of pancreatic ductal adenocarcinoma reveals antigen-presenting cancer-associated fibroblasts. Cancer Discov. **9**(8), 1102–1123 (2019) 31197017 10.1158/2159-8290.CD-19-0094 PMC6727976

[17] G. Caligiuri, D.A. Tuveson, Activated fibroblasts in cancer: Perspectives and challenges. Cancer Cell. **41**(3), 434–449 (2023) 36917949 10.1016/j.ccell.2023.02.015 PMC11022589

[18] L. Milane, A. Singh, G. Mattheolabakis, M. Suresh, M.M. Amiji, Exosome mediated communication within the tumor microenvironment. J. Contr. Release. **219**, 278–294 (2015) 10.1016/j.jconrel.2015.06.029 26143224

[19] A. Becker, B.K. Thakur, J.M. Weiss, H.S. Kim, H. Peinado, D. Lyden, Extracellular vesicles in cancer: Cell-to-cell mediators of metastasis. Cancer Cell. **30**(6), 836–848 (2016) 27960084 10.1016/j.ccell.2016.10.009 PMC5157696

[20] N. Kosaka, Decoding the secret of cancer by means of extracellular vesicles. J. Clin. Med. **5**2, (2016) 10.3390/jcm5020022 PMC4773778 26861408

[21] J.L. Hu, W. Wang, X.L. Lan, Z.C. Zeng, Y.S. Liang, Y.R. Yan, et al., CAFs secreted exosomes promote metastasis and chemotherapy resistance by enhancing cell stemness and epithelial-mesenchymal transition in colorectal cancer. Mol. Cancer **18**(1), 91 (2019) 31064356 10.1186/s12943-019-1019-x PMC6503554

[22] Y. Gao, X. Li, C. Zeng, C. Liu, Q. Hao, W. Li, et al. CD63(+) cancer-associated fibroblasts confer tamoxifen resistance to breast cancer cells through exosomal miR-22. Adv. Sci (Weinh) **7**(21), 2002518 (2020) 10.1002/advs.202002518 PMC7610308 33173749

[23] T. Fang, H. Lv, G. Lv, T. Li, C. Wang, Q. Han, et al., Tumor-derived exosomal miR-1247-3p induces cancer-associated fibroblast activation to foster lung metastasis of liver cancer. Nat. Commun. **9**(1), 191 (2018) 29335551 10.1038/s41467-017-02583-0 PMC5768693

[24] B. Chen, Y. Sang, X. Song, D. Zhang, L. Wang, W. Zhao, et al., Exosomal miR-500a-5p derived from cancer-associated fibroblasts promotes breast cancer cell proliferation and metastasis through targeting USP28. Theranostics **11**(8), 3932–47 (2021) 10.7150/thno.53412 PMC7914354 33664871

[25] T. Liu, C. Han, P. Fang, Z. Ma, X. Wang, H. Chen, et al., Cancer-associated fibroblast-specific lncRNA LINC01614 enhances glutamine uptake in lung adenocarcinoma. J. Hematol. Oncol. **15**(1), 141 (2022) 36209111 10.1186/s13045-022-01359-4 PMC9548164

[26] R. Qi, Y. Bai, K. Li, N. Liu, Y. Xu, E. Dal, et al., Cancer-associated fibroblasts suppress ferroptosis and induce gemcitabine resistance in pancreatic cancer cells by secreting exosome-derived ACSL4-targeting miRNAs. Drug Resist. Updat.. **68**, 100960 (2023) 37003125 10.1016/j.drup.2023.100960

[27] L. Liu, H. Huang, B. Cheng, H. Xie, W. Peng, H. Cui, et al., Revealing the role of cancer-associated fibroblast senescence in prognosis and immune landscape in pancreatic cancer. iSci. **28**(1), 111612 (2025) 10.1016/j.isci.2024.111612 PMC11742819 39834857

[28] W. Chen, W. Peng, R. Wang, S. Bai, M. Cao, S. Xiong, et al., Exosome-derived tRNA fragments tRF-GluCTC-0005 promotes pancreatic cancer liver metastasis by activating hepatic stellate cells. Cell Death Dis. **15**(1), 102 (2024) 38291031 10.1038/s41419-024-06482-3 PMC10827722

[29] B. Adem, N. Bastos, C.F. Ruivo, S. Sousa-Alves, C. Dias, P.F. Vieira, et al., Exosomes define a local and systemic communication network in healthy pancreas and pancreatic ductal adenocarcinoma. Nat. Commun. **15**(1), 1496 (2024) 38383468 10.1038/s41467-024-45753-7 PMC10881969

[30] W. Chen, W. Peng, R. Wang, S. Bai, M. Cao, S. Xiong, et al., Exosome-derived tRNA fragments tRF-GluCTC-0005 promotes pancreatic cancer liver metastasis by activating hepatic stellate cells. Cell. Death Dis. **15**(1), 102 (2024) 38291031 10.1038/s41419-024-06482-3 PMC10827722

[31] X. Wang, R. Wang, S. Bai, S. Xiong, Y. Li, M. Liu, et al., Musashi2 contributes to the maintenance of CD44v6+ liver cancer stem cells via notch1 signaling pathway. J. Exp. Clin. Cancer Res. **38**(1), 505 (2019) 31888685 10.1186/s13046-019-1508-1 PMC6936093

[32] S. Roessler, H.L. Jia, A. Budhu, M. Forgues, Q.H. Ye, J.S. Lee, et al., A unique metastasis gene signature enables prediction of tumor relapse in early-stage hepatocellular carcinoma patients. Cancer Res. **70**(24), 10202–10212 (2010) 21159642 10.1158/0008-5472.CAN-10-2607 PMC3064515

[33] S.M. Kim, I.-S. Leem Sh Fau - Chu, -Y.-Y. Chu Is Fau - Park, S.C. Park Yy Fau - Kim, S.-B. Kim Sc Fau - Kim, E.S. Kim Sb Fau - Park, et al., Sixty-five gene-based risk score classifier predicts overall survival in hepatocellular carcinoma. Hepatol. **55**(5) (2012) 10.1002/hep.24813 PMC4060518 22105560

[34] D. Zeng, Z. Ye, R. Shen, G. Yu, J. Wu, Y. Xiong, et al., IOBR: Multi-omics immuno-oncology biological research to decode tumor microenvironment and signatures. Front. Immunol. **12**, 687975 (2021) 34276676 10.3389/fimmu.2021.687975 PMC8283787

[35] Y. Sun, L. Wu, Y. Zhong, K. Zhou, Y. Hou, Z. Wang, et al., Single-cell landscape of the ecosystem in early-relapse hepatocellular carcinoma. Cell **184**(2), 404–21e16 (2021) 33357445 10.1016/j.cell.2020.11.041

[36] T. Wu, E. Hu, S. Xu, M. Chen, P. Guo, Z. Dai, et al., clusterProfiler 4.0: A universal enrichment tool for interpreting omics data. Innov. (Camb.) **2**(3), 100141 (2021) 10.1016/j.xinn.2021.100141 PMC8454663 34557778

[37] Y. Yan, H. Tao, J. He, S.-Y. Huang, The HDOCK server for integrated protein–protein docking. Nat. Protoc.. **15**(5), 1829–1852 (2020) 32269383 10.1038/s41596-020-0312-x

[38] T. Hou, J. Wang, Y. Li, W. Wang, Assessing the performance of the MM/PBSA and MM/GBSA methods. 1. The accuracy of binding free energy calculations based on molecular dynamics simulations. J. Chem. Inf. Model. **51**(1), 69–82 (2011) 21117705 10.1021/ci100275a PMC3029230

[39] L. Han, E.W. Lam, Y. Sun, Extracellular vesicles in the tumor microenvironment: Old stories, but new tales. Mol. Cancer **18**(1), 59 (2019) 30925927 10.1186/s12943-019-0980-8 PMC6441234

[40] F. Wen, Y. Han, H. Zhang, Z. Zhao, W. Wang, F. Chen, et al., Epstein-barr virus infection upregulates extracellular OLFM4 to activate YAP signaling during gastric cancer progression. Nat. Commun. **15**(1), 10543 (2024) 39627192 10.1038/s41467-024-54850-6 PMC11615309

[41] Q. Shi, C. Xue, Y. Zeng, X. Yuan, Q. Chu, S. Jiang, et al., Notch signaling pathway in cancer: From mechanistic insights to targeted therapies. Signal Transduct. Target. Ther. **9**(1), 128 (2024) 38797752 10.1038/s41392-024-01828-x PMC11128457

[42] S. Kaufhold, B. Fau - Bonavida, B. Bonavida, Central role of Snail1 in the regulation of EMT and resistance in cancer: A target for therapeutic intervention. J. Exp. Clin. Cancer Res. **33**(1), 62 (2014) 10.1186/s13046-014-0062-0 PMC4237825 25084828

[43] A. Vogel, T. Meyer, G. Sapisochin, R. Salem, A. Saborowski, Hepatocellular carcinoma. The Lancet** 400**(10360), 1345–62 (2022) 10.1016/S0140-6736(22)01200-4 36084663

[44] J.D. Yang, P. Hainaut, G.J. Gores, A. Amadou, A. Plymoth, L.R. Roberts, A global view of hepatocellular carcinoma: trends, risk, prevention and management. Nat. Rev. Gastroenterol. Hepatol.. **16**(10), 589–604 (2019) 31439937 10.1038/s41575-019-0186-y PMC6813818

[45] K. Nakamura, M.J. Smyth, Myeloid immunosuppression and immune checkpoints in the tumor microenvironment. Cell Mol. Immunol. **17**(1), 1–12 (2020) 31611651 10.1038/s41423-019-0306-1 PMC6952382

[46] T. Tang, X. Huang, G. Zhang, Z. Hong, X. Bai, T. Liang, Advantages of targeting the tumor immune microenvironment over blocking immune checkpoint in cancer immunotherapy. Signal Transduct. Target. Ther. **6**(1), 72 (2021) 33608497 10.1038/s41392-020-00449-4 PMC7896069

[47] G. Ishii, A. Ochiai, S. Neri, Phenotypic and functional heterogeneity of cancer-associated fibroblast within the tumor microenvironment. Adv Drug Deliv Rev. **99**,186–96 (2016) 10.1016/j.addr.2015.07.007 26278673

[48] H. Xu, J. Zhao, J. Li, Z. Zhu, Z. Cui, R. Liu, et al., Cancer associated fibroblast-derived CCL5 promotes hepatocellular carcinoma metastasis through activating HIF1α/ZEB1 axis. Cell Death Dis. **13**(5), 478 (2022) 10.1038/s41419-022-04935-1 PMC9119971 35589690

[49] H. Wang, Z. Lu, X. Zhao, Tumorigenesis, diagnosis, and therapeutic potential of exosomes in liver cancer. J. Hematol. Oncol. **12**(1), 133 (2019) 31815633 10.1186/s13045-019-0806-6 PMC6902437

[50] P. Zheng, Q. Luo, W. Wang, J. Li, T. Wang, P. Wang, et al., Tumor-associated macrophages-derived exosomes promote the migration of gastric cancer cells by transfer of functional apolipoprotein E. Cell Death Dis. **9**(4), 434 (2018) 29567987 10.1038/s41419-018-0465-5 PMC5864742

[51] L. Kular, J. Pakradouni, P. Kitabgi, M. Laurent, C. Martinerie, The CCN family: A new class of inflammation modulators? Biochimie **93**(3), 377–388 (2011) 21130134 10.1016/j.biochi.2010.11.010

[52] F. Hall-Glenn, K.M. Lyons, Roles for CCN2 in normal physiological processes. Cell Mol. Life Sci. **68**(19), 3209–3217 (2011) 21858450 10.1007/s00018-011-0782-7 PMC3670951

[53] Y.W. Shen, Y.D. Zhou, H.Z. Chen, X. Luan, W.D. Zhang, Targeting CTGF in cancer: An emerging therapeutic opportunity. Trends Cancer **7**(6), 511–524 (2021) 33358571 10.1016/j.trecan.2020.12.001

[54] T.T. Wang, J.H. Yuan, J.Z. Ma, W.J. Yang, X.N. Liu, Y.P. Yin, et al., CTGF secreted by mesenchymal-like hepatocellular carcinoma cells plays a role in the polarization of macrophages in hepatocellular carcinoma progression. Biomed. Pharmacother. **95**, 111–119 (1950-6007 (Electronic)) (2017) 10.1016/j.biopha.2017.08.004 28837877

[55] M. Seehawer, Z. Li, J. Nishida, P. Foidart, A.H. Reiter, E. Rojas-Jimenez, et al., Loss of Kmt2c or Kmt2d drives brain metastasis via KDM6A-dependent upregulation of MMP3. Nat. Cell. Bio. **26**(7), 1165–1175 (2024) 38926506 10.1038/s41556-024-01446-3 PMC11251985

[56] T. Masuda, A. Fukuda, G. Yamakawa, M. Omatsu, M. Namikawa, M. Sono, et al., Pancreatic RECK inactivation promotes cancer formation, epithelial-mesenchymal transition, and metastasis. J. Clin. Invest. **133**(18), (2023) 10.1172/JCI161847 PMC10503799 37712427

[57] J. Liang, M. Chen, D. Hughes, A.A. Chumanevich, S. Altilia, V. Kaza, et al., CDK8 selectively promotes the growth of colon cancer metastases in the liver by regulating gene expression of timp3 and matrix metalloproteinases. Cancer Res. **78**(23), 6594–6606 (2018) 30185549 10.1158/0008-5472.CAN-18-1583 PMC6279600

[58] Y.-X. Li, -X.-X. Zhu, X. Wu, J.-H. Li, X.-H. Ni, S.-J. Li, et al., ACLP promotes activation of cancer-associated fibroblasts and tumor metastasis via ACLP-PPARγ-ACLP feedback loop in pancreatic cancer. Cancer Lett. **544**, 215802 (2022) 35732215 10.1016/j.canlet.2022.215802

[59] B. Liu, T. Liu, Y. Liu, X. Feng, X. Jiang, J. Long, et al., TSG-6 promotes cancer cell aggressiveness in a CD44-dependent manner and reprograms normal fibroblasts to create a pro-metastatic microenvironment in colorectal cancer. Int. J. Biol. Sci. **18**(4), 1677–1694 (2022) 35280699 10.7150/ijbs.69178 PMC8898369

[60] W. Guo, B. Zhou, L. Dou, L. Guo, Y. Li, J. Qin, et al., Single-cell RNA sequencing and spatial transcriptomics of esophageal squamous cell carcinoma with lymph node metastases. Exp. Mol. Med. **57**(1), 59–71 (2025) 39741182 10.1038/s12276-024-01369-x PMC11799171

[61] C. Luo, H. Xin, Z. Zhou, Z. Hu, R. Sun, N. Yao, et al., Tumor-derived exosomes induce immunosuppressive macrophages to foster intrahepatic cholangiocarcinoma progression. Hepatol. **76**(4), 982–999 (2022) 10.1002/hep.32387 35106794

[62] R. Jackstadt, S.R. van Hooff, J.D. Leach, X. Cortes-Lavaud, J.O. Lohuis, R.A. Ridgway, et al., Epithelial NOTCH signaling rewires the tumor microenvironment of colorectal cancer to drive poor-prognosis subtypes and metastasis. Cancer Cell **36**(3), 319–36e7 (2019) 31526760 10.1016/j.ccell.2019.08.003 PMC6853173

[63] A.O. Rehman, C.Y. Wang, Notch signaling in the regulation of tumor angiogenesis. Trends Cell Biol. **16**(6), 293–300 (2006) 16697642 10.1016/j.tcb.2006.04.003

[64] O. Meurette, P. Mehlen, Notch signaling in the tumor microenvironment. Cancer Cell **34**(4), 536–548 (2018) 10.1016/j.ccell.2018.07.009 30146333

[65] V. Bolos, M. Blanco, V. Medina, G. Aparicio, S. Diaz-Prado, E. Grande, Notch signalling in cancer stem cells. Clin. Transl. Oncol. **11**(1), 11–19 (2009) 19155199 10.1007/s12094-009-0305-2

[66] J.Y. Lee, S.Y. Song, J.Y. Park, Notch pathway activation is associated with pancreatic cancer treatment failure. Pancreatology **14**(1), 48–53 (2014) 10.1016/j.pan.2013.11.011 24555978

[67] A. Leonetti, F. Facchinetti, R. Minari, A. Cortellini, C.D. Rolfo, E. Giovannetti, et al., Notch pathway in small-cell lung cancer: From preclinical evidence to therapeutic challenges. Cell Oncol. (Dordr) **42**(3), 261–273 (2019) 30968324 10.1007/s13402-019-00441-3 PMC12994342

[68] O. Kranenburg, Prometastatic NOTCH signaling in colon cancer. Cancer Discov. **5**(2), 115–117 (2015) 25656897 10.1158/2159-8290.CD-14-1456

[69] M. Jin, J. Wang, X. Ji, H. Cao, J. Zhu, Y. Chen, et al., MCUR1 facilitates epithelial-mesenchymal transition and metastasis via the mitochondrial calcium dependent ROS/Nrf2/notch pathway in hepatocellular carcinoma. J. Exp. Clin. Cancer Res. **38**(1), 136 (2019) 30909929 10.1186/s13046-019-1135-x PMC6434841

[70] S. Fang, M. Liu, L. Li, F.F. Zhang, Y. Li, Q. Yan, et al., Lymphoid enhancer-binding factor-1 promotes stemness and poor differentiation of hepatocellular carcinoma by directly activating the NOTCH pathway. Oncogene **38**(21), 4061–4074 (2019) 30696957 10.1038/s41388-019-0704-y

[71] J.H. Lee, S.M. Jung, K.M. Yang, E. Bae, S.G. Ahn, J.S. Park, et al., A20 promotes metastasis of aggressive basal-like breast cancers through multi-monoubiquitylation of Snail1. Nat. Cell. Bio. **19**(10), 1260–73 (2017) 10.1038/ncb3609 28892081

[72] M. Qiu, D. Chen, C. Shen, J. Shen, H. Zhao, Y. He, Sex-determining region Y-box protein 3 induces epithelial-mesenchymal transition in osteosarcoma cells via transcriptional activation of Snail1. J. Exp. Clin. Cancer Res.. **36**(1), 46 (2017) 28335789 10.1186/s13046-017-0515-3 PMC5364714

[73] F. Li, H. Zhao, M. Su, W. Xie, Y. Fang, Y. Du, et al., HnRNP-F regulates EMT in bladder cancer by mediating the stabilization of Snail1 mRNA by binding to its 3’ UTR. EBioMedicine **45**, 208–219 (2352-3964 (Electronic)) (2019) 10.1016/j.ebiom.2019.06.017 PMC6642227 31221586

[74] E. Canalis, S. Zanotti, A. Smerdel-Ramoya, Connective tissue growth factor is a target of notch signaling in cells of the osteoblastic lineage. Bone **64**, 273–280 (2014) 24792956 10.1016/j.bone.2014.04.028 PMC4069863

[75] J. Luo, H. Xu, C. Su, W. Dong, M. Xiao, N. Xiao, et al., Polo-like kinase2 regulates renal tubulointerstitial fibrosis via notch signaling pathway in diabetic kidney disease. Faseb. **39**(5), (2025) 10.1096/fj.202402793R PMC11891471 40059448

[76] A. Mazzocca, E. Fransvea, F. Dituri, L. Lupo, S. Antonaci, G. Giannelli, Down-regulation of connective tissue growth factor by inhibition of transforming growth factor beta blocks the tumor-stroma cross-talk and tumor progression in hepatocellular carcinoma. Hepatol. **51**(2), 523–534 (2010) 10.1002/hep.23285 19821534

[77] B. Chen, X. Liu, P. Yu, F. Xie, J.S.H. Kwan, W.N. Chan, et al., H. pylori-induced NF-κB-PIEZO1-YAP1-CTGF axis drives gastric cancer progression and cancer-associated fibroblast-mediated tumour microenvironment remodelling. Clin. Trans. Med. **13**(11), (2023) 10.1002/ctm2.1481 PMC10659770 37983931

