# Supplementary material for: Cancer-associated fibroblast-derived extracellular vesicles facilitate metastasis in hepatocellular carcinoma by delivering CTGF
Source: Cell Oncol (Dordr). 2025 Jul 1;48(5):1413–32. doi: 10.1007/s13402-025-01085-2 (PMC12528347; doi:10.1007/s13402-025-01085-2)
Supplement: Supplementary file 1 — Supplementary Material 1 [file 13402_2025_1085_MOESM1_ESM.docx]

**Supplementary information**

**Cancer-associated fibroblast-derived extracellular vesicles facilitate metastasis in hepatocellular carcinoma by delivering CTGF**

**Journal name: Cellular Oncology**

Mengli Zheng^1,3,*^, Luyao Liu^1,*^, Haochen Cui^1^, Yuchong Zhao^1^, Wei Chen^1^, Shuya Bai^1^, Wang Peng^1^, Yun Wang^1^, Yanling Li^1^, Ronghua Wang^4^, Xiju Wang^1,2,#^, Bin Cheng^1,#^

^1^ Department of Gastroenterology and Hepatology, Tongji Hospital, Tongji Medical College, Huazhong University of Science and Technology, Wuhan 430030, China.

^2^ Department of Digestive Endoscopy, The Affiliated Hospital of Guizhou Medical University, Guiyi Street No. 28, Guiyang, Guizhou, China 550000.

^3^ Department of Gastroenterology, The Tenth Affiliated Hospital of Southern Medical University (Dongguan People's Hospital), Southern Medical University, Dongguan 523000, Guangdong Province, China.

^4^ Department of Surgery, University of Pittsburgh School of Medicine, Pittsburgh, PA, 15213, USA.

^*^ Mengli Zheng and Luyao Liu contributed equally to this work.

**^#^ Corresponding Author:** Bin Cheng, Department of Gastroenterology and Hepatology, Tongji Hospital, Tongji Medical College, Huazhong University of Science and Technology, Wuhan 430030, China. E-mail: [b.cheng@tjh.tjmu.edu.cn](mailto:b.cheng@tjh.tjmu.edu.cn). Xiju Wang, Department of Digestive Endoscopy, The Affiliated Hospital of Guizhou Medical University, Guiyi Street No. 28, Guiyang, Guizhou, China 550000. E-mail: 920168029@qq.com

**Supplementary figures**

**Fig. S1**

**
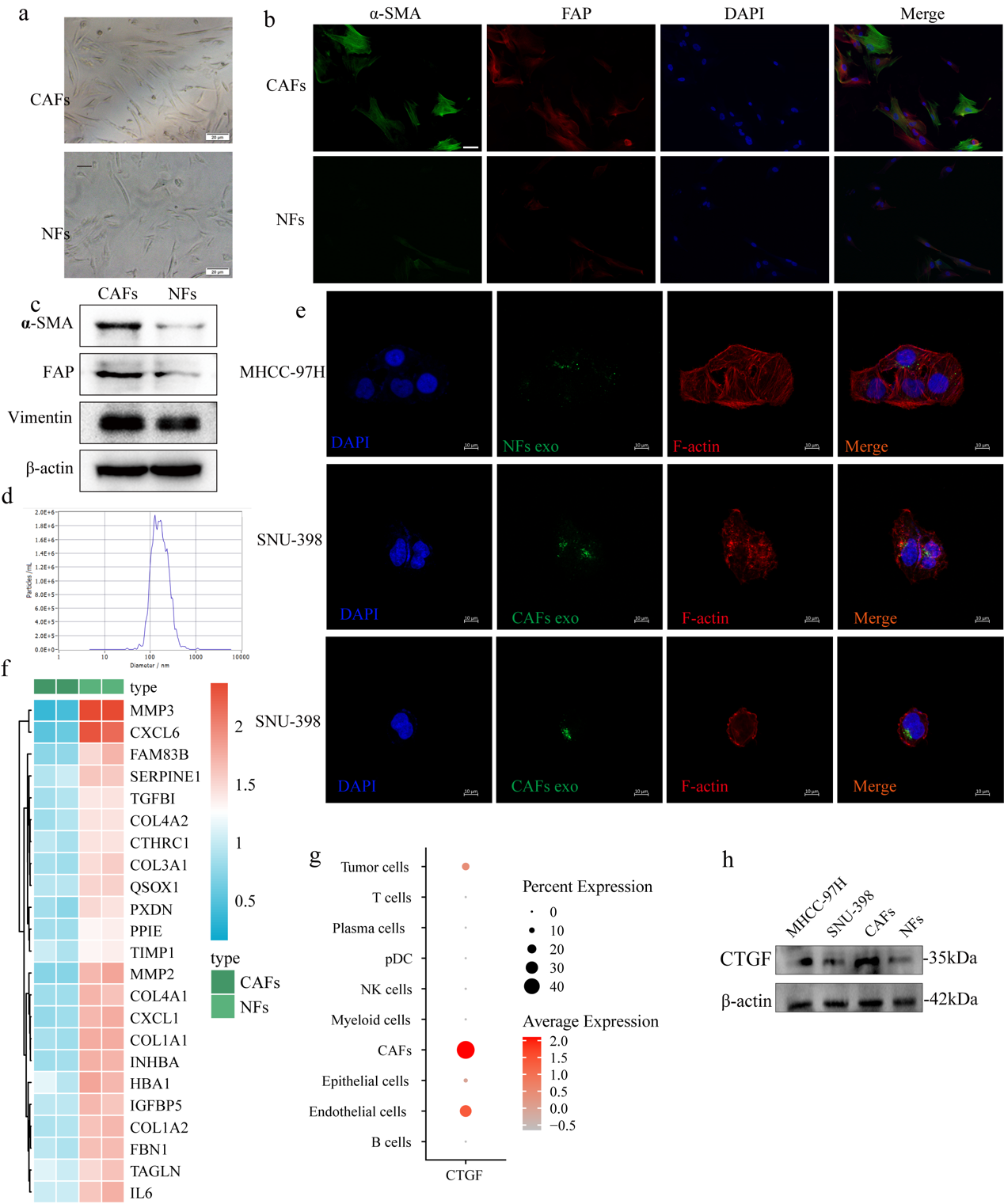
**

**a.** The morphology of CAFs and NFs (Scale bar, 20 μm). **b.** α-SMA, FAP, and vimentin levels were assessed by IF staining in CAFs and NFs (Scale bar, 20 μm). **c**. α-SMA, FAP, and vimentin levels were assessed by western blot in CAFs and NFs. **d.** Nanoparticle tracking analysis of NFs-EV. **e.** Uptake of PKH67-labeled CAFs-EV and NFs-EV by MHCC-97H and SNU-398 cells observed via confocal microscopy (Scale bar, 10 μm). **f.** Heat map of upregulated proteins in NFs-EV compared to CAFs-EV. **g.** The expression levels of CTGF in various cell types in the TME of HCC. **h.** The expression levels of CTGF in CAFs, NFs, and HCC cells.

**Fig. S2**

**
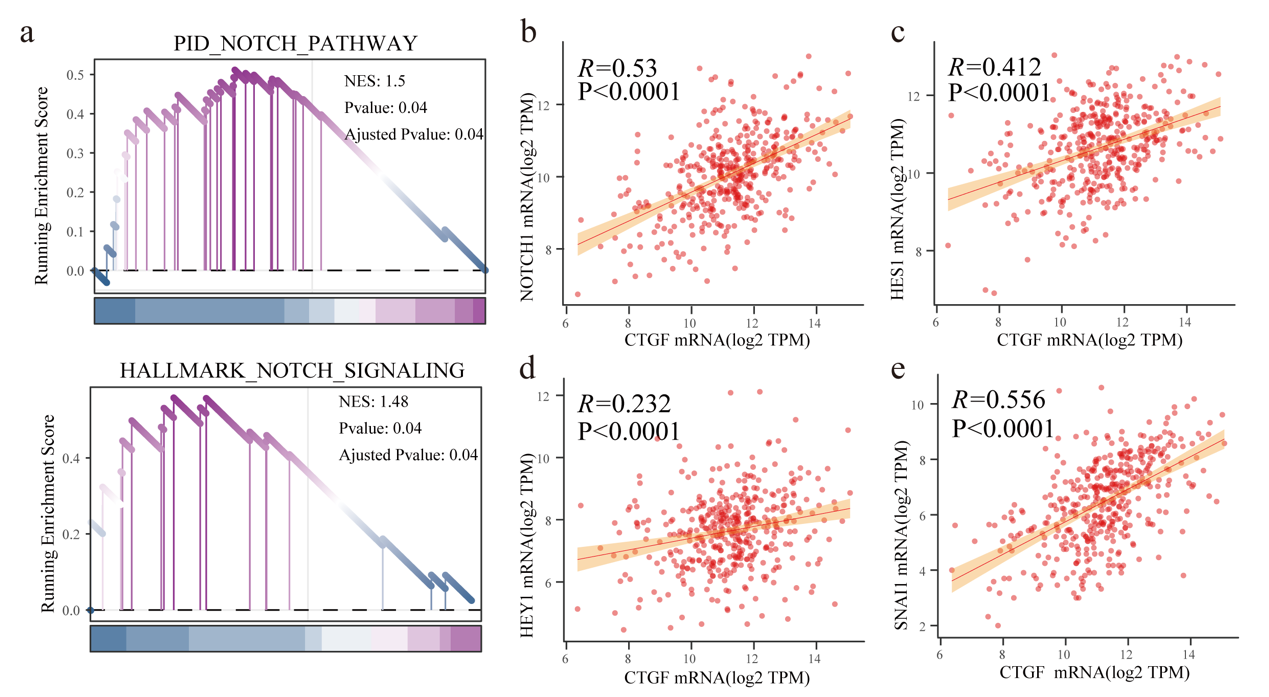
**

**a.** GSEA analysis showed enrichment of the Notch signaling pathway in the CTGF^high^ group. **b-d.** Relationship between CTGF expression and Notch1 pathway genes, NOTCH1 (**b**), HES1 (**c**), and HEY1 (**d**) expression. **e.** Relationship between CTGF expression and Snail1 expression.

**Fig. S3**

**
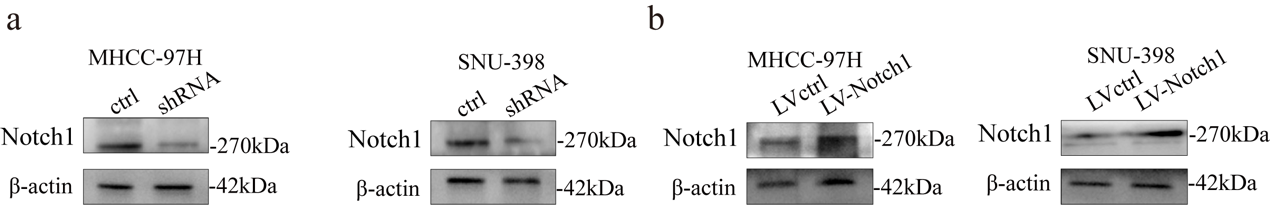
**

**a.** Notch1 knockdown efficiency was validated by western blot in MHCC-97H and SNU-398 cells. **b.** Notch1 overexpression efficiency was validated by western blot in MHCC-97H and SNU-398 cells.

**Fig. S4**

**
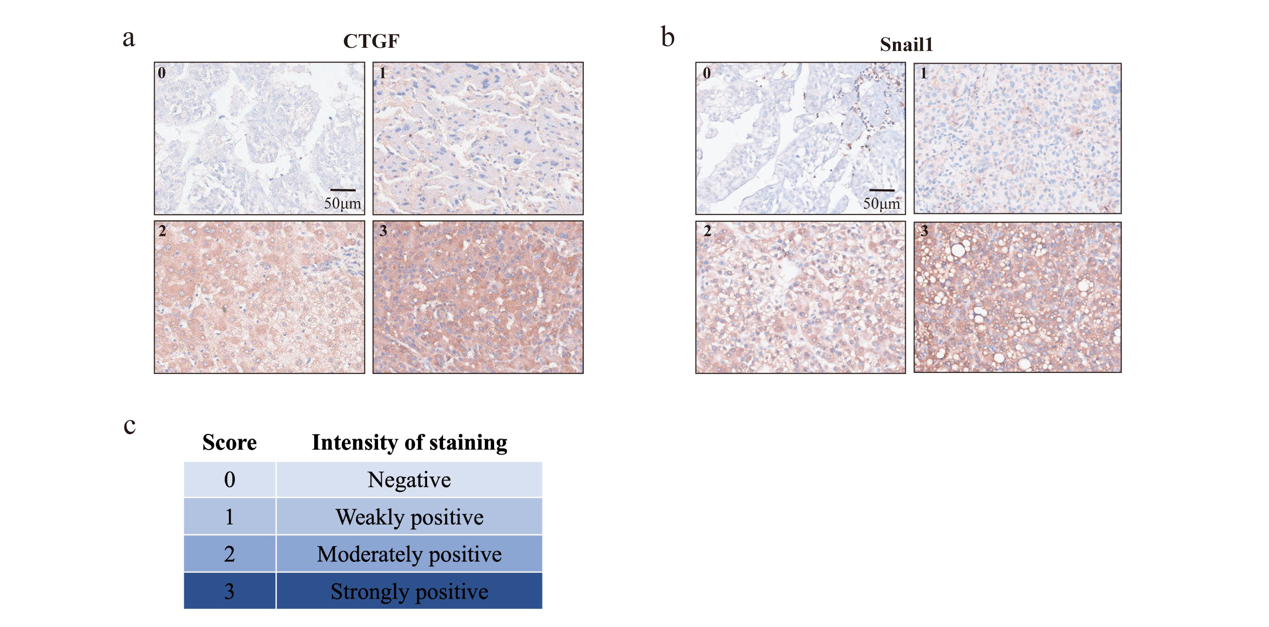
**

**a-c.** IHC staining for CTGF (**a**) and Snail1 (**b**) and the intensity of staining (**c**) were evaluated as described in the methods. For each marker, representative images were shown demonstrating each of the intensity grades of staining (Scale bar, 50 μm).

**Supplementary tables**

**Table. S1 Antibodies used in this study**

| **Protein name** | **Host species** | **Product number** | **dilutions** |
| --- | --- | --- | --- |
| WB antibody | | | |
| a-SMA | M | BOSTER, BM0002 | 1:1000 |
| FAP | M | Abnova, H00002191-M01 | 1:1000 |
| Vimentin | R | ABGENT, AX1005 | 1:1000 |
| Notch1 | R | Proteintech, 20687-1-ap | 1:600 |
| NICD | M | Abcam, ab52627 | 1:2000 |
| HES-1 | R | Abclonal, A11718 | 1:1000 |
| HEY-1 | R | Proteintech,19929-1-ap | 1:1000 |
| Snail1 | R | Abclonal, A11794 | 1:1000 |
| CTGF | R | Abclonal, A11067 | 1:1000 |
| CD9 | R | Abclonal, A19027 | 1:1000 |
| CD81 | R | Abclonal, A4863 | 1:1000 |
| CD63 | R | Abclonal, A19023 | 1:1000 |
| TSG101 | R | Abclonal, A1692 | 1:1000 |
| β-actin | R | Servicebio, GB11001-100 | 1:2000 |
|  | | | |
| IF antibody | | | |
| a-SMA | M | BOSTER, BM0002 | 1:200 |
| FAP | M | Abnova, H00002191-M01 | 1:200 |
| CTGF | R | Abclonal, A11067 | 1:200 |
| Notch1 | R | Proteintech, 20687-1-ap | 1:200 |
| Alexa Fluor488 Goat anti-R IgG | G | Servicebio, Gb25303 | 1:500 |
| Alexa Fluor488 Goat anti-M IgG | G | Servicebio, Gb25301 | 1:500 |
| Cyanine3 Goat anti-R IgG | G | Servicebio, Gb21303 | 1:500 |
| Cyanine3 Goat anti-M IgG | G | Servicebio, Gb31301 | 1:500 |
|  |  |  |  |
| IHC antibody |  |  |  |
| CTGF | R | Abclonal, A11067 | 1:200 |
| Snail1 | R | Abclonal, A11794 | 1:200 |
| HRP-Goat anti-R IgG | G | Servicebio, G1213-100UL | 1:200 |

**Table. S2 The sequences of shRNA**

| **Primer or virus** | **sequence** |
| --- | --- |
| CTGF shRNA | shRNA Ctrl, 5′- CCTAAGGTTAAGTCGCCCTCG-3′ |
|  | shRNA CTGF, 5’- CCGACTGGAAGACACGTTT-3’ |
| Notch1 shRNA | shRNA Ctrl, 5′- CCTAAGGTTAAGTCGCCCTCG-3′ |
|  | shRNA Notch1, 5’- TGGCGGGAAGTGTGAAGCG-3’ |
